# Exploring Winegrowers’ Behaviours and Ecological Impacts Under Climate Change and Policy Scenarios—Examples from Three European Winegrowing Regions

**DOI:** 10.1007/s00267-023-01924-8

**Published:** 2024-01-11

**Authors:** Yang Chen, Stefan Möth, Silvia Winter, Louise Willemen, Nina Schwarz

**Affiliations:** 1 Faculty of Geo-Information Science and Earth Observation, University of Twente, Hallenweg 8, 7522 NH Enschede, The Netherlands; 2 Governance and Inclusive Development, Faculty of Social and Behavioral Sciences, University of Amsterdam, New Achtergracht 166, 1001 NC Amsterdam, The Netherlands; 3 Department of Crop Sciences, Institute of Plant Protection, University of Natural Resources and Life Sciences, Vienna, Gregor-Mendel-Straße 33, 1180 Vienna, Austria

**Keywords:** Agent-based model, Agricultural policy, Locally adapted models, Pesticides, Sustainable viticulture, Climate change

## Abstract

Viticulture is an example of a socio-ecological system that poses serious challenges for sustainable soil management and pesticide use, with various interactions between winegrowers’ decision-making and ecological consequences. This study introduces an agent-based model (ABM) on winegrowers’ decision on inter-row management and pesticide use. The ABM builds upon an empirical study of winegrowers’ decision-making in European viticultural landscapes and has been built for three case study regions: Leithaberg (Austria), Palatinate (Germany) and Târnave (Romania). The ABM allows for analysing potential effects of policy instruments including mandatory vegetation cover in the inter-rows, the reduction of fungicide use and ban of insecticides against *Lobesia botrana*. The effects of policies differ between the case study regions, indicating how important the local context is for effective policies. For example, policies aiming at higher inter-row vegetation cover had the strongest effects on vegetation cover, landscape aesthetics and soil loss in Târnave since many vineyards are currently intensively tilled and there exist no policies supporting inter-row vegetation cover in Romania.

## Highlights

- We introduce an agent-based model suite for three European winegrowing regions.
- The model explores vegetation and pest management and their ecological consequences.
- The feedback loop between decision-making and ecological processes is closed.
- Each region is simulated under climate change and various policy instruments.
- Results show that context is important for effective policies.

## Introduction

Effective management and policy-making of complex socio-ecological systems require an understanding of decision-making processes and how human behaviours affect the biophysical environment (Elsawah et al. 2015). The understanding of decision-making in socio-ecological systems has been advanced by a number of recent studies, for example, on farmers’ adoption of sustainable behaviours (Bartkowski and Bartke 2018; Dessart et al. 2019). As knowledge of decision-making processes in socio-ecological systems improves, there is also need to advance the models used to simulate what-if scenarios of policies and their potential impacts (Schlüter et al. 2017). Decision-making processes need to consider dispositional, social, and cognitive factors to explicitly represent heterogeneity (Bartkowski and Bartke 2018; Dessart et al. 2019; Huber et al. 2018). Also, simulating the interactions between human behaviour and their ecological consequences remains a challenge (Filatova et al. 2013). Without accounting for these feedbacks, socio-ecological simulation models fall short of a comprehensive representation of the targeted systems and, thus, are not suitable for investigating solutions for sustainability challenges (Everard 2020).

Viticulture is a typical example of a socio-ecological system whose soil management and pesticide use may support or threaten sustainable land use (Paiola et al. 2020). Moreover, viticulture needs to respond to climate change (Santos et al. 2020) and policies aiming at reducing pesticide use (European Commission 2020) and higher vegetation cover (Winter et al. 2018) Viticultural landscapes do not only support the livelihoods of winegrowers and their cultural traditions but also provide various ecosystem services and may conserve biodiversity (Winkler et al. 2017; Winter et al. 2018). In these landscapes, winegrowers respond to changes of the climatic conditions by regulating the vines and the soils, which influences pests, their predators, and fungal diseases, resulting in various human-nature interactions over time. Particularly important for biodiversity and ecosystem services are two sets of practices: inter-row management and pesticide use (Nascimbene et al. 2013; Paredes et al. 2021; Winter et al. 2018). Winegrowers’ choices of vegetation cover vs. bare soil in the inter-rows do not only affect soil erosion mitigation in vineyards (Biddoccu et al. 2020) but also lead to different pest-predator dynamics that either reduce or increase the need for winegrowers’ pesticide use (Blaise et al. 2022; Möth et al. 2023). The presence of vegetation in the inter-rows can provide habitats and food resources for natural enemies that prey on vine pests, but such benefit needs to be weighed by winegrowers against their concerns regarding the competition of vegetation and vines for water and nutrients (Pardini et al. 2002; Ripoche et al. 2011; Siepmann and Nicholas 2018). Winegrowers’ pesticide use is mainly preventive due to the large potential damage of pests and diseases (such as fungicide use against mildew diseases or insecticide use against grape berry moth, for the latter pheromone dispenser instead of insecticides could also be used for mating interference). However, the potential negative environmental effects of pesticide use are increasingly criticised by the public.

At the European scale, winegrowers’ behaviours and their ecological impacts differ from one region to another in response to different climatic and edaphic conditions: from hot and dry Mediterranean Spanish vineyards over temperate oceanic climate in German vineyards to temperate continental climate in Austrian and Romanian vineyards (Biddoccu et al. 2020). In addition, various socio-economic and political factors differ in these regions that may interact and affect winegrowers’ practices (Chen et al. 2022) and consequently their ecological impacts such as soil loss, pest abundance, biological pest control, biodiversity, and landscape aesthetics. Simulation models can explore how well policies perform given these heterogeneities and complex relationships between human behaviours and associated ecological processes. Such simulation models need to account for heterogeneity between viticultural landscapes and winegrowers to include winegrowers as agents of decision-making.

Agent-based models (ABMs) are well suited for modelling human behaviours based on heterogeneity of agents. Despite the fast-growing numbers of ABMs for farmers’ behaviour and agriculture in general (An 2012; Huber et al. 2018), their applications in viticulture are still at an early stage. Lammoglia et al. (2019) provide an overview of models applied to viticulture. Various behaviours of winegrowers are represented in these ABMs, such as land use choices between vines and other food crops (Delay et al. 2017), decisions to remain or leave a winegrowing cooperative (Delay et al. 2015), decisions on the number of vine plots to be cultivated in different terrain characteristics (Zottele and Delay 2014), the organisation of viticultural practices that are constrained by the limited resources of the winegrower (Martin-Clouaire et al. 2016), and winegrowers’ inter-row management and pesticide use (Tissot et al. 2017). The majority of these models assume utility maximising on the decision-making process and usually do not consider any ecological consequences of the modelled behaviour, nor do they include adaptation of behaviours to changing environmental factors. One exception is the SEVE model (Tissot et al. 2017), in which the simulation of the decision-making process of winegrowers includes the relationships between vine phenology, winegrowers’ practices and the adaptation to climate and disease factors. Most of the models mentioned above are designed for or applied to one specific case-study region, and none investigates the effects of policies on different viticultural regions.

This study aims to fill this research gap by constructing a series of ABMs which simulate not only winegrowers’ behaviours but also their ecological impacts for three European winegrowing regions, namely Leithaberg in Austria, Palatinate in Germany and Târnave in Romania. With the developed ABMs, we aim to answer the following research question: How do the different viticultural landscapes evolve under climate change and policy instruments? The two sub-questions are: (1) how do winegrowers’ behaviours and related ecological impacts evolve in the three case studies with and without climate change? (2) How do policies influence ecological impacts and potential adaptations of winegrowers’ other behaviours (not targeted by the policy) in the three case studies?

## Model Development, Simulation and Analyses

**Fig. 1:**
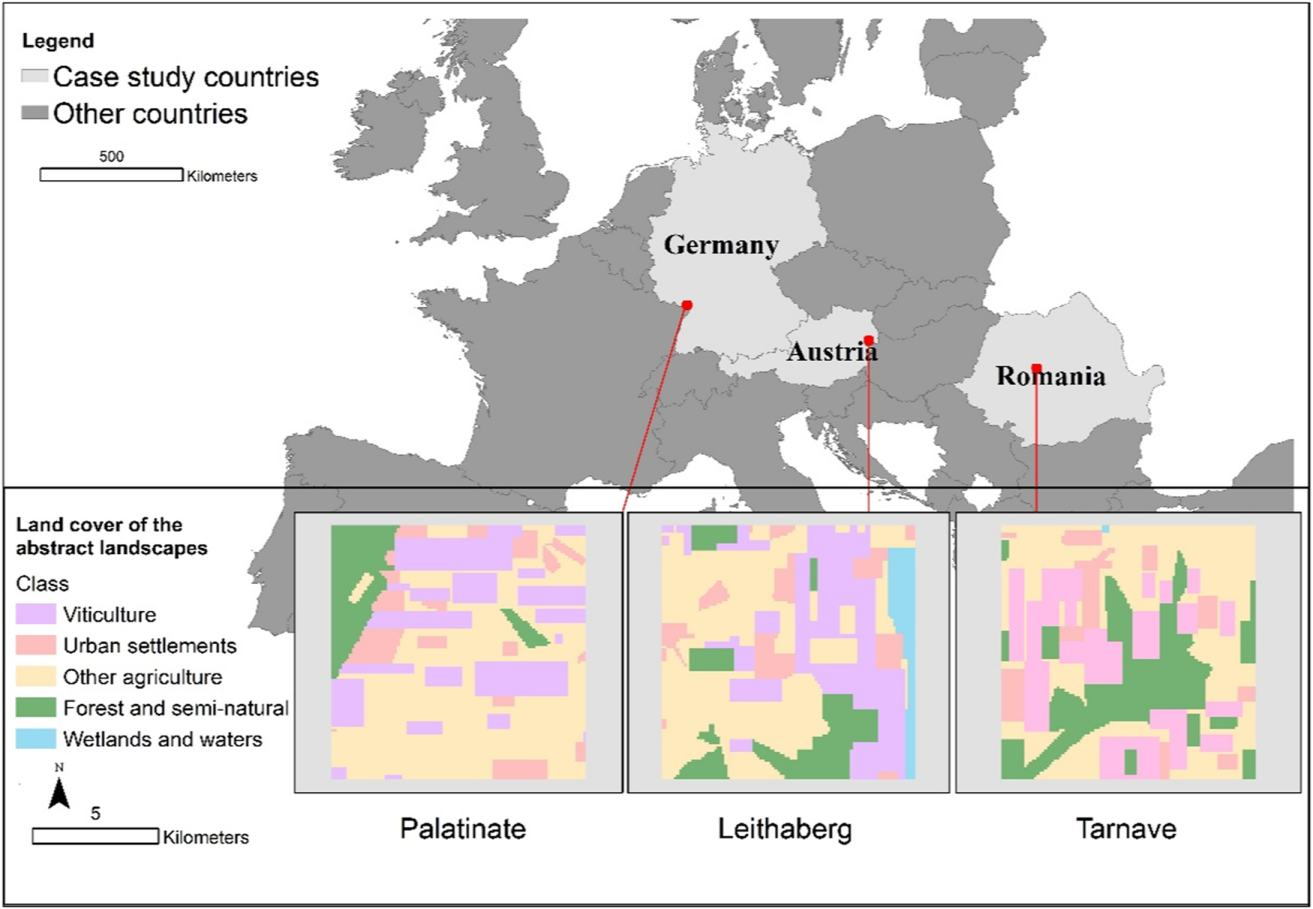
Locations of the three case studies and the abstract landscapes in each of the three regions. The main map shows the regions (indicated as red) where an abstract landscape (10 km × 10 km) was created based on the CORINE land cover map (2018). Each of these abstract landscapes was used to set up the space of the corresponding ABM

Our simulation study builds upon an earlier empirical study of winegrowers’ decision-making in European viticultural landscapes (Chen et al. 2022). We now developed for three case studies an agent-based model in which human-nature interactions are represented to explore how the system evolves under climate change and policy scenarios: Leithaberg in Austria, Palatinate in Germany and Târnave in Romania (Fig. 1). This is a convenience sample focussing on the case studies of Chen et al. (2022) with sufficient data (adequate survey responses from which statistically significant relationships between winegrower and vineyard characteristics can be derived) on winegrowers’ decision-making to build an ABM.

### Overall Design for the Model Development: From Concept to Implementation

**Fig. 2:**
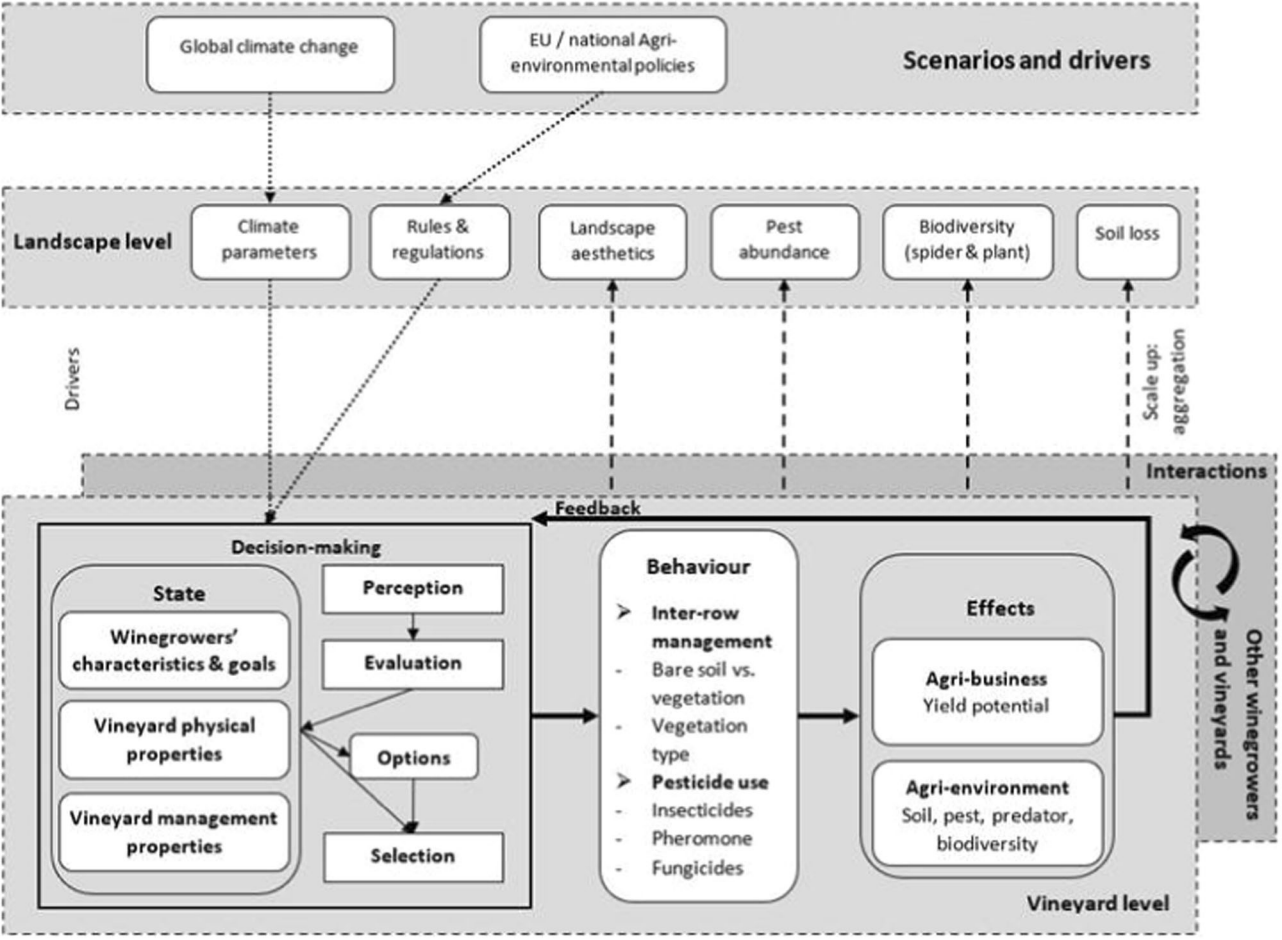
Our conceptual model to explore winegrowers’ inter-row management and pesticide use and their ecological impacts across European viticultural landscapes

All three case studies are simulated with the same ABM which can be initialised for each case study. The detailed description of the model, following the ODD (Overview, Design Concepts, and Details) + D (Decision) protocol—a standard for describing ABMs that include human decision making (Müller et al. 2013), is provided in Appendix 1. The model itself and the documentation is available at https://www.comses.net/codebases/7922d774-be9c-4719-9342-8f5d4db866c6/releases/1.0.0/. Overall, the ABM is based on the following conceptual considerations (see Fig. 2):

1. At the vineyard level, winegrowers decide autonomously on their inter-row management (bare soil vs. inter-row vegetation, and if so vegetation in every vs. every 2nd inter-row) and pesticide use (insecticides, pheromones, and/or fungicides). Their behaviours have consequences for grapevine yield potentials, soil loss, pest abundance, pest control (by natural predators), and biodiversity (plants and spiders). Over time, these ecosystem services feed back to winegrowers’ decision-making, which is also affected by climate change and different policies. Depending on winegrowers’ management, personal characteristics, and physical properties of the vineyard, they respond to these changes and adapt their behaviours accordingly. During their adaptation to conditions on their own vineyard and to newly introduced policies, winegrowers may interact with others, for example, by observing the dominant behaviour within the landscape.
2. At the landscape level, scenarios regarding climatic conditions (higher or lower temperature, and more or less precipitation) and policies may affect winegrowers’ decision-making, for example, a new rule acting as constraints for their management options and climatic conditions as triggers of their adaptation behaviours. Also, at this level, aggregations of vineyard-level ecological impacts resulting from their behaviours are determined to describe, for example, landscape aesthetics, average soil loss and biodiversity.
3. At the highest level are the processes of global climate change and EU and national agri-environmental policy-making. They determine in the targeted landscapes what climatic conditions and policies are likely to affect each landscape and the winegrowers.

Based on the conceptual model, we implemented the ABM as follows:

1. For each case study, winegrowers’ behaviours were initialised by using the decision trees that were empirically tested (see “Winegrowers’ characteristics and decision rules derived from empirical evidence”).
2. A generic rule of behavioural change for winegrowers was implemented to update their behaviours during the simulation (see “Rules for behaviour change of winegrowers”).
3. For each case study, a viticultural landscape was generated to represent the spatial configuration of the modelled system as taken from CORINE land cover (Corine 2018) (see “Landscape configuration”).
4. Winegrowers’ behaviours and their ecological impacts were coupled by using published empirical findings and data analyses provided by experts (see “Linking behaviours with ecological impacts”).
5. Finally, external drivers of the modelled system included predicted climatic parameters and policy scenarios (see “Climate scenario design” and “Policy scenarios”).

Finally, we conducted interviews with winegrowers to verify the rules of behavioural change and asked local experts to validate the modelled behavioural statistics.

#### Winegrowers’ characteristics and decision rules derived from empirical evidence

Heterogeneity of winegrowers is reflected in different initial values for winegrowers’ characteristics such as vineyard size and management type (see Appendix 1 section I.ii.b). These values are informed by the empirical study on winegrowers’ decision-making (Chen et al. 2022). This empirical study also includes a range of behaviours concerning inter-row management and pesticide use (Chen et al. 2022). We included the following behaviours in the ABM: (1) arrangement of inter-row vegetation—bare soil vs. vegetation in every 2nd inter-row vs. vegetation in every inter-row, (2) inter-row vegetation type—no vegetation vs. spontaneous vegetation vs. seed mixture, (3) insecticide use—binary and if yes, the annual frequency, (4) pheromone dispenser use—binary, and (5) annual fungicide frequency including synthetic fungicides, copper- or sulfur-based fungicides. The empirical study derived decision trees for each case study (Chen et al. 2022) based on average 33 surveys of winegrowers, and these were used to set up the initial behaviours of winegrowers in the ABM.

#### Rules for behaviour change of winegrowers

The complete set of rules of behaviour change are described in Appendix 2. Winegrowers’ behaviours have ecological consequences and their future behaviours are also affected by climate change and different policies, but in different ways:

- For any newly introduced policy, winegrowers are aware of the regulation before it is implemented and such regulation will affect the behavioural options from the time it is implemented.
- For climatic conditions, winegrowers are sensing changes in temperature and precipitation “on-the-fly” and adapt their behaviours (mostly concerning the use of insecticides and fungicides) accordingly.
- For ecological consequences such as changes in yield, soil erosion, and biodiversity, winegrowers reflect on these results and deliberate a change in their behaviour, should one or more of them become a concern.

#### Landscape configuration

To effectively communicate the model and model results to winegrowers and also to allow winegrowers from each case study to identify and refer to their local environment, a spatially explicit representation is needed. For each case study (Leithaberg, Palatinate, and Târnave), a 10 km × 10 km landscape from the corresponding region is imported to the ABM, based on the CORINE Land Cover Map (2018). These landscapes contain the land use/cover types of urban settlements, viticulture, other types of agriculture, forests, semi-natural elements, and wetlands/water. The share of the viticultural area in each landscape is similar (26–27%). In order to avoid identification of specific locations and undesired personal attachment to these locations from winegrowers to whom the ABM is presented, we abstracted each landscape so that the share of different land use/cover roughly remained but the shape of these elements was altered and simplified. The detailed translation of these landscapes can be found in Appendix 1. However, each vineyard is visualised in the model interface as an elongated rectangle, which represent their shapes in reality. Additionally, characteristics such as vineyard sizes, slope, problems with water shortage and soil are all generated randomly at the individual level, with the scaled-up statistics at the landscape level in correspondence with empirical data. The ability of the model to represent vineyards and the local environment in a spatially explicit way allows us to incorporate local interactions such as neighbourhood effects. Such interactions were not implemented in the model due to a lack of support from our empirical evidence. Nevertheless, the spatially explicit model proved useful in model communication and has the potential for further development.

#### Linking behaviours with ecological impacts

**Fig. 3:**
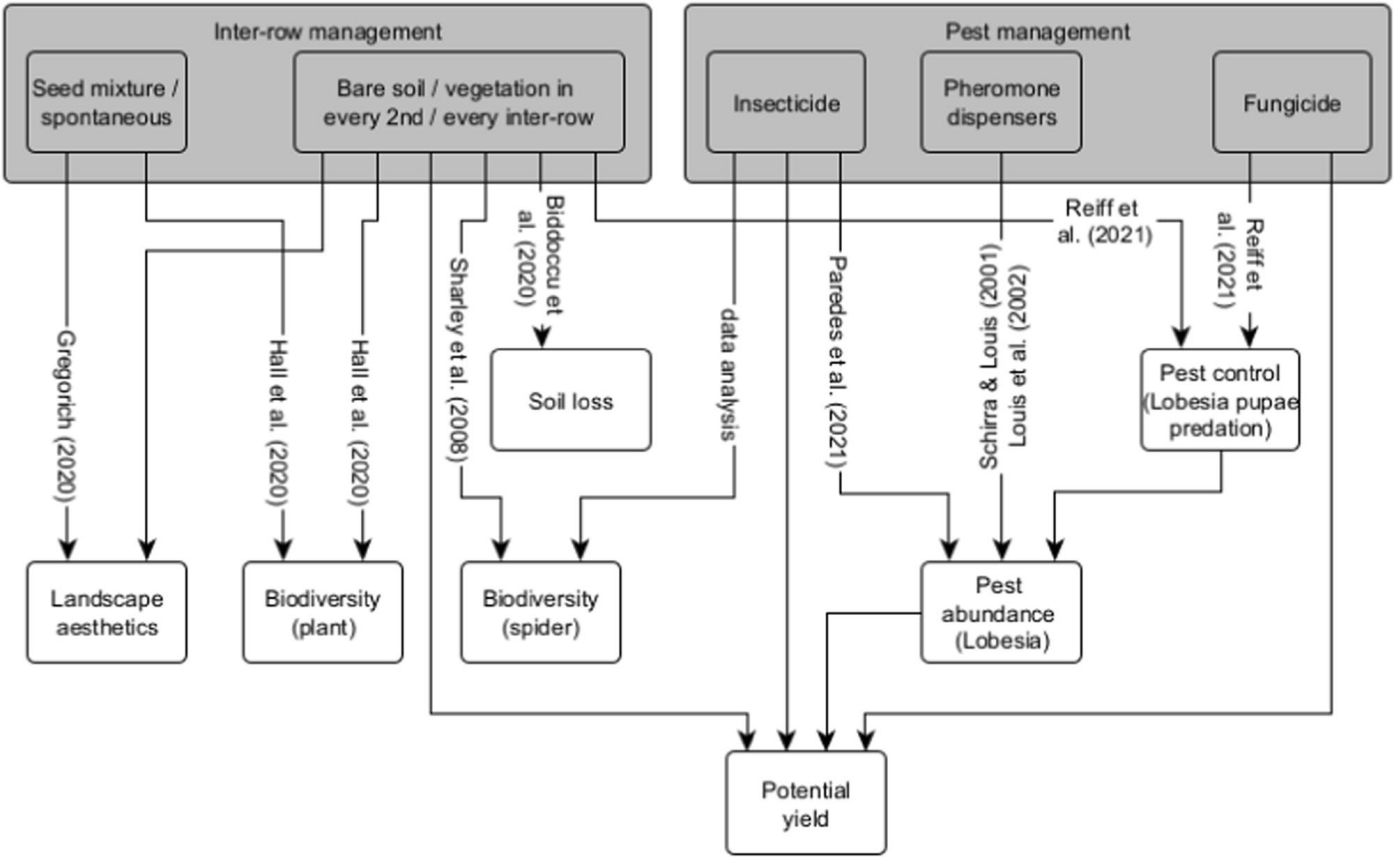
Causal relationships between winegrowers’ behaviours and their ecological impacts. The rectangles in grey are modelled behaviours and the rest are the ecological variables, the links represent casual relationships, with quantitative values found in literature or data analyses. Note that landscape aesthetics is only quantified as the share of vegetated inter-rows for the whole landscape and all links to potential yield are implemented as either “to increase” or “to decrease” (see Appendix 1) without exact quantities due to a lack of literature and the possible pre-harvest yield reduction behaviour to achieve higher quality wines

Winegrowers’ behaviours are connected to their ecological consequences on ecosystem services such as yield potential, landscape aesthetics and natural pest control, and on ecosystem disservices such as pest abundance focusing on *Lobesia botrana* (Denis and Schiffermüller), soil loss as well as vascular plant and spider diversity. In our model, we used quantitative relationships based on parameter values in published articles (Biddoccu et al. 2020; Gregorich 2020; Hall et al. 2020; Louis et al. 2002; Paredes et al. 2021; Reiff et al. 2021; Schirra and Louis 2001; Sharley et al. 2008) and data analyses from natural scientists in field studies of the research project SECBIVIT. These relationships are visualised in Fig. 3. If empirical data pointed to context-specific ecological relationships, different parameters for each case study were used. Otherwise, the same relationship was used across all cases. For details, see also Appendix 1.

#### Climate scenario design

Europe is projected to be warmer but with varying precipitation patterns for different regions (Ljungqvist et al. 2019). For simplicity, only one climate change scenario was used in addition to the current conditions. This scenario is defined by temperature and precipitation relative to the current conditions: is it warmer or colder, and is it drier or wetter. Based on Ljungqvist et al. (2019) and climate monitoring of the relevant case studies (meteoblue 2022a–c), changes in the mean yearly temperature and precipitation, both relative to the current conditions, are used to define a climate change scenario for the next 10 years. The qualitative differences are used instead of the exact quantity of temperature and precipitation anomaly because winegrowers reported their adaptative behaviours referring to such qualitative terms. The temperature scenario describes the future with years to be warmer and years to be a lot warmer; and the precipitation scenario describes the future with wetter years, drier years, and normal years with regard to precipitation. In the implemented climate change scenario, there are 5 years that are much warmer than normal years, 3 years of wetter, 4 years of drier, and 3 years of normal precipitation conditions (see Table 1). Besides the climate change scenario, we also implemented a scenario in which temperature and precipitation are assumed to be identical with the current situation (no climate change) as a reference.

**Table 1:** Climate change scenario design as input for the model

Climate variable (annual)	Design of the climate parameters for future 10 years (left to right: 2022–2031)									
Temperature	1	2	1	2	1	2	1	2	1	2
Precipitation	1	−1	1	0	1	−1	0	−1	−1	0

Annual mean temperatures are predicted to be warmer (1: warmer and 2: a lot warmer), and annual mean precipitations are predicted to fluctuate (1: wetter, 0: no change, and −1: drier)

#### Policy scenarios

The European Union has envisioned a more sustainable agricultural sector via the Green Deal (European Commission 2019). This would mean vineyard inter-rows with vegetation cover and less pesticide use for viticulture (2030 goal: 50% reduction in pesticide use). We considered in total seven policy scenarios (including a no new policy scenario) that regulate either inter-row management or pesticide use in European viticultural landscapes (Table 2). In each scenario, 100% implementation is assumed, so the respective behaviour is fixed while all other behaviours including adapting to impacts of these policies are not.

**Table 2:** Policy scenarios as input for the model

ID	Policy scenario	Implementation in the model
1-NA	No new policy	
2-CS	Inter-row vegetation: must be implemented in vineyards on slope. Due to the concern of erosion, vegetation has to be in every inter-row.	Find vineyards with average slope >10%, mark these vineyards with obligation, change inter-row in these vineyards in the next year after the policy. In the check options part, mark these winegrowers and do not allow removing vegetation cover. When winegrowers can choose to have vegetation in every or every 2nd inter-row, they do it randomly. When the affected winegrowers used to have only bare soil in the inter-rows, the vegetation type they adopt is based on the dominant type of inter-row cover at the landscape scale.
3-CW	Inter-row vegetation: must be installed in vineyards with no water shortage problem. Vegetation can be in every inter-row or every 2nd.	Find vineyards with water shortage problem = false, and the rest actions are as same as in 2-CS.
4-CA	Inter-row vegetation: must be installed in all vineyards. Vegetation can be in every inter-row or every 2nd.	Find all vineyards, and the rest actions are as same as in 2-CS.
5-NI	Insecticide: must not be used	Find vineyards with insecticide >0, mark these vineyards with obligation, change insecticide use in next year to 0. In the check options part, mark all winegrowers and remove the option of increasing insecticide use.
6-PO	Pheromone dispenser: must be used	Find vineyards with pheromone = no, mark these vineyards with obligation, change use in next year to yes.
7-FR	Fungicide frequency: must be reduced by one application	Select all winegrowers, mark them with obligation, reduce total fungicide applications in the next year by one application. In the check options part, mark all winegrowers and remove the option of increasing fungicide use.

Note that only one policy scenario is implemented per model run

### Model Experimental Design, Selected Measurements of Model Outcomes and Analyses

Our ABMs (one for each case study) are based on empirical evidence of the current situation and are used to explore future scenarios. To avoid increasing uncertainties due to processes that are not included in our conceptual model (such as competition between viticulture and other forms of agriculture, inter-generational transfer and change of vineyard management, etc.), we limit the time horizon of a model run to 10 years, with one time step simulating 1 year. To focus on the (accumulated) effects, all policy scenarios (except the one without any new policy) are implemented in the beginning of a model run. At the end of a model run, a number of measurements were taken (see “Selected model outcomes”); model outcomes were compared with expert’s knowledge for validation, visualisation and analyses, and further interpretation for policy implications (see “Validation and sensitivity analysis”).

#### Selected model outcomes

The model outcomes reported for this study are provided in Table 3. Note that the ABMs produce more outcomes than what are listed here. For example, the “Share of vineyards with vegetation in every 2nd inter-row” can be derived from the first and second indicator, and the “share of inter-row vegetation as spontaneous vegetation” can be derived from the third indicator in Table 3.

**Table 3:** Model outcomes on winegrowers’ behaviours and their ecological impacts

Type	Indicators	Aggregation
Winegrowers’ behaviours	Share of vineyards with vegetation in every inter-row	Mean of all time steps
	Share of vineyards with bare soil only inter-rows	
	Share of inter-row vegetation as seed mixture	
	Mean annual insecticide frequency	
	Share of vineyards using pheromone dispensers	
	Mean annual frequency of copper- and/or sulfur-based fungicides	
	Mean annual frequency of synthetic fungicide	
Ecological impacts	Landscape aesthetics: area extent of vegetated inter-rows at the landscape scale (for the whole case study region)	
	Mean annual soil loss of vineyards	
	Mean vascular plant diversity of vineyards	
	Mean spider diversity of vineyards	
	Mean predation rate of *L. botrana* pupae	
	Share of vineyards with high *L. botrana* abundance	
	Total times (events) yield potentials are increased per vineyard	End of model run
	Total times (events) yield potentials are decreased per vineyard	

#### Validation and sensitivity analysis

An et al. (2021) distinguish four types of validation: input validation, process validation, descriptive output validation and predictive output validation. Documenting and reflecting the data origin, quality and limitations of the survey as the basis of the agents’ decision-making is covered in detail in a separate publication (Chen et al. 2022). Data to parameterise the ecological processes were taken from existing literature and were not independently validated. For process validation, we thoroughly discussed the choices on entities and processes included and left out in our project team. The reasoning is documented in the ODD protocol and not further elaborated here. Instead, we focus here on the comparison of model outputs regarding winegrowers’ behaviour with estimates provided by local experts. These estimates were provided before the validation runs were conducted. However, we already had impressions on differences between the case studies from our field visits, so our validation seems to fall between the descriptive output validation (i.e., comparison with data used to build the model) and prescriptive output validation (i.e., comparison with independent and previously unknown data).

Model outcomes were qualitatively validated with information from local experts from the research team SECBIVIT. To support the validation process, we sent questionnaire surveys to experts from each case study region and collected expert estimates on winegrowers’ behaviours, prior to the generation of any model outcomes. These experts were researchers who have worked intensively with winegrowers from the respective regions, details are provided in Appendix 3. We asked their estimates (average and if possible, also the range) of winegrowers’ behaviour. We then compared modelled outcomes (concerning winegrowers’ behaviours) against expert estimates based on ten model runs for each case study without additional policies and no climate change. Modelled outcomes were considered to agree with experts’ statements if the modelled average fell within the range provided by the local experts or close to the range. Missing expert values for Palatinate were substituted with those for Leithaberg. We do not have ecological data for all case studies to validate the modelled ecological impacts.

We also conducted one-factor-at-a-time (OFAT) sensitivity analysis on some key model parameters where empirical data and/or literature was limited. These model parameters concerned yield potentials, soil loss and biodiversity to which winegrowers respond, the starting time of a policy, etc. Results suggested that there were no issues of strong sensitivity in these parameters and therefore we presented model outcomes in the remainder of this study by using reference parameter values (Appendix 1).

#### Experimental design

Model outcomes were compared across case studies and policy scenarios. For a unique parameter configuration, the ABM was run 10 times to account for stochasticity. A repetition of 10 per parameter configuration is a typical ABM practice (Ben-Dor et al. 2021; Bichraoui-Draper et al. 2015; Koch et al. 2019) for models that are not very sensitive to initial conditions, which our model was found to be the case. This also allowed us to allocate more computation power to focus on a wide range of scenarios. However, we recognise it as a convenient model design choice and are aware that with more model complexity probably more repetitions are required (Troost et al. 2023). To investigate the effect of climate change, or a specific policy on the model outcomes, we conducted unpaired two-sample Wilcoxon tests for significance testing.

## Results

### Overview of Model Outcome Validation

Appendix 3 summarises the validation results for all the modelled outcomes without policies. Out of the 21 outcomes of winegrowers’ behaviours (seven indicators across three case studies), 11 were found to lie within the expected range provided by local experts, five were close to that, and only five were clearly outside of the expected range. “Share of inter-row vegetation as seed mixture” was found to be underestimated for Leithaberg, as local experts reported 85% vineyards being seeded with cover crop mixtures while the ABM showed around 52%. So was “share of vineyards using pheromone dispensers” for Leithaberg and Târnave: the former was found to be underestimated (57.6% instead of reported 90%) by the model and the latter overestimated (62.6% instead of reported 25%). Insecticide use was overestimated by our model for Tarnave (7.1 instead of 3 applications per year). Consequently, plant diversity was probably underestimated for Leithaberg vineyards due to lower simulated seed mixture use, vineyards with high *L. botrana* abundance was probably overestimated for Leithaberg vineyards and underestimated for Târnave vineyards because of misrepresentation of pheromone dispenser use, and spider diversity was probably underestimated for Târnave vineyards due to the overestimation of their insecticide use. The rest of the model outcomes represented the realities of each case study well enough We did not modify the model based on the validation findings but kept these shortcomings in mind when interpreting the results of the policy scenarios.

**Fig. 4:**
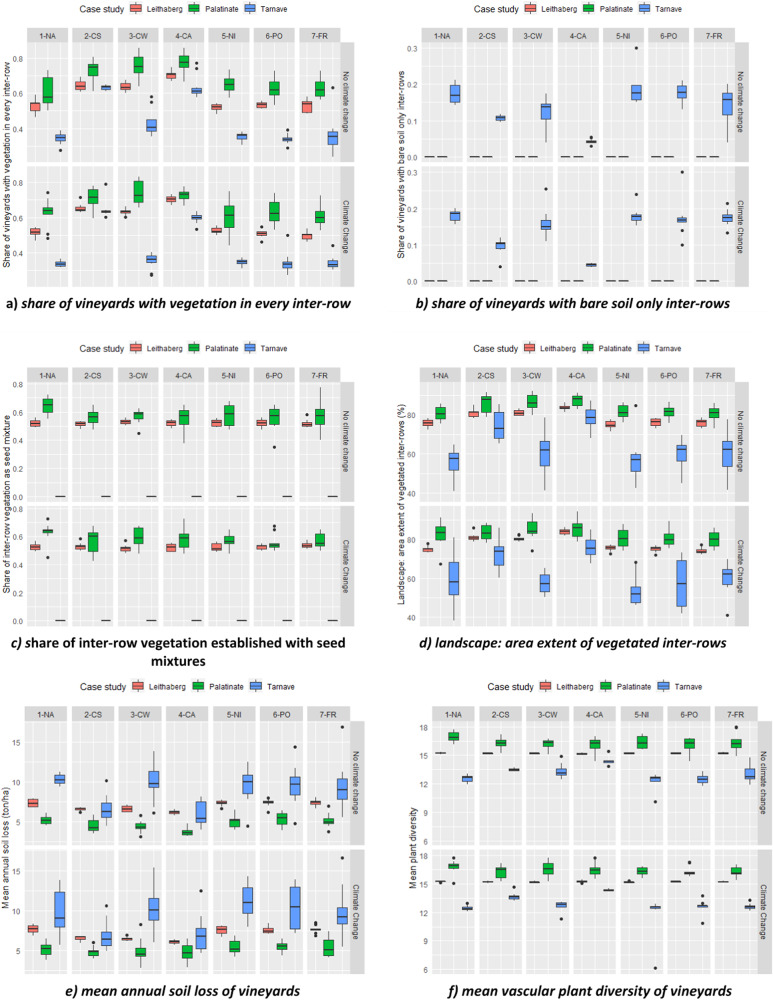
**a**–**f** Simulated effects of policy scenarios and climate change on winegrowers’ inter-row management and consequent ecological impacts. 1-NA: no policy; 2-CS: compulsory inter-row vegetation if situated on slope; 3-CW: compulsory if no water shortage; 4-CA: compulsory on all; 5-NI: No insecticide allowed; 6-PO: pheromone obligation; 7-FR: fungicide reduction. For details on the policies, see Table 2. The right-hand panel indicates if climate change is considered. **a** Share of vineyards with vegetation in every inter-row; **b** share of vineyards with bare soil only inter-rows; **c** share of inter-row vegetation established with seed mixtures; **d** landscape: area extent of vegetated inter-rows; **e** mean annual soil loss of vineyards; **f** mean vascular plant diversity of vineyards

**Fig. 5:**
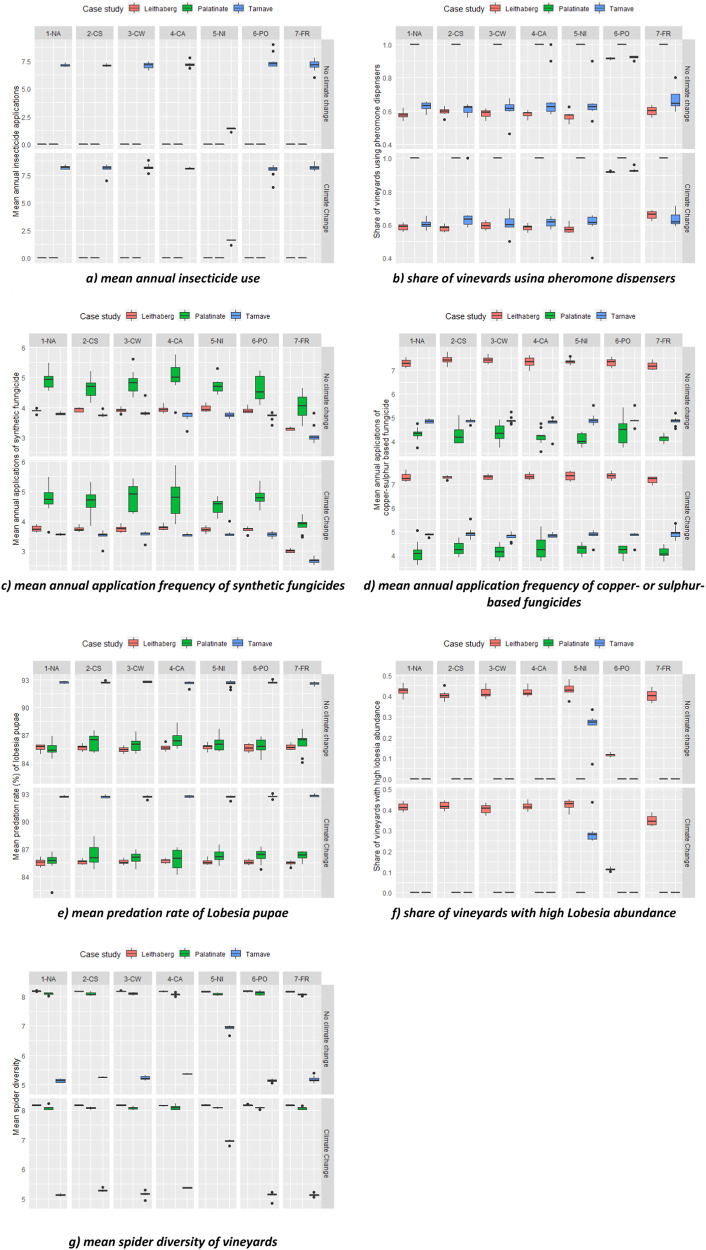
**a**–**g** Simulated effects of policy scenarios and climate change on winegrowers’ pest management use and consequent ecological impacts. Note that inter-row management can also influence (**e**) and (**g**). 1-NA: no policy; 2-CS: compulsory inter-row vegetation if situated on slope; 3-CW: compulsory if no water shortage; 4-CA: compulsory on all; 5-NI: No insecticide allowed; 6-PO: pheromone obligation; 7-FR: fungicide reduction. For details on the policies, see Table 2. The right-hand panel indicates if climate change is considered. **a** Mean annual insecticide use; **b** share of vineyards using pheromone dispensers; **c** mean annual application frequency of synthetic fungicides; **d** mean annual application frequency of copper- or sulfur-based fungicides; **e** mean predation rate of Lobesia pupae; **f** share of vineyards with high Lobesia abundance; **g** mean spider diversity of vineyards

We used model runs in which neither climate change nor any policy scenario was implemented as references. In the reference scenario (upper left panel in Figs. 4 and 5), we found the following across the regions. First, Leithaberg and Palatinate vineyards have “greener” inter-rows than Târnave vineyards—the former two showed a higher share of vegetation in every inter-row (Fig. 4a), no vineyards with bare soil in the inter-rows (Fig. 4b), and higher share of seed mixture as inter-row vegetation type (Fig. 4c). Consequently, vineyards in Leithaberg and Palatinate were found to have higher landscape aesthetics (Fig. 4d), lower soil loss (Fig. 4e), and higher plant diversity (Fig. 4f). Second, insecticides are only used in Târnave (Fig. 5a), pheromone dispenser use was much higher in Palatinate compared to Târnave and Leithaberg (Fig. 5b). Palatinate winegrowers sprayed more frequently synthetic fungicides while Leithaberg winegrowers sprayed more frequently with copper- or sulfur-based fungicides (Fig. 5c, d), *L. botrana* pupae predation rate was highest in Târnave (Fig. 5e), Leithaberg had (likely overestimated, see above) higher *L. botrana* abundance (Fig. 5f), and spider diversity was lowest in Târnave (Fig. 5g).

### Effects of Climate Change on Behaviours and Related Ecological Impacts Across Case Studies

With no new policy considered (1-NA), we compared the simulated effects of climate change on winegrowers’ behaviours and related ecological impacts (Figs. 4 and 5). While regional differences are clear in Figs. 4 and 5, our model simulated no significant changes (*p* = 0.01 level) in winegrowers’ behaviours and ecological impacts under climate change, except for the following: increase of insecticide use in Târnave (Fig. 5a) and decrease of synthetic fungicide in Leithaberg and Târnave (Fig. 5c) were found as simulated effects of climate change (for details see Appendix 4).

### Effects of Policy Scenario on Behaviours and Ecological Impacts Across Case Studies

Figure 4 summarises model outcomes of winegrowers’ inter-row management behaviours and related ecological consequences under different policies (1-NA to 7-FR) and climate change scenarios. For each model outcome, the reference scenario is at the top left indicating no new policy and no climate change. In the following, we report on all statistically significant relationships, including the immediate effects of policies on related behaviours.

Policies have mixed effects on inter-row management and associated ecological consequences. Compared against results in 1-NA (no policy change) without climate change, we found the following statistically significant effects: (1) inter-row related policies 2-CS, 3-CW, and 4-CA all increased the share of vineyards with vegetation in every inter-row in all case studies (Fig. 4a); (2) accordingly the same policies also decreased the share of vineyards with bare soil inter-rows in Târnave (Fig. 4b) and increased the overall share of vegetated inter-rows at the landscape scale in all case studies (Fig. 4d), except for a statistically non-significant effect for 3-CW in Târnave; (3) winegrowers’ use of seed mixtures for establishing vegetation in the inter-rows was rather unaffected by all policies in all case studies except for policies 2-CS, 3-CW, and 4-CA in Palatinate, where these policies significantly decreased seed mixture use (Fig. 4c); (4) the policies 2-CS, 3-CW and 4-CA significantly decreased soil loss in Leithaberg, Palatinate (except for 2-CS), and Târnave (except for 3-CW), see Fig. 4e; (5) plant diversity responded differently across the case studies to policies 2-CS, 3-CW and 4-CA—diversity significantly decreased in Palatinate but increased in Târnave, and was not affected in Leithaberg (Fig. 4f); and there were (6) no clear effect of policies 5-NI, 6-PO, and 7-FR on inter-row related behaviours and related ecological consequences, except for a significant decrease of the use of seed mixtures caused by 6-PO in Palatinate (Fig. 4c). Statistical tests of the comparisons can be found in Appendix 4.

Figure 5 summarises model outcomes of winegrowers’ pest management and related ecological consequences under different policies (1-NA to 7-FR) and climate scenarios. Compared against the reference scenario (1-NA, no climate change), each of the pesticide-specific policy scenarios was found to have the expected effect. For example, policy 5-NI significantly reduced the insecticide use in Târnave (there was no insecticide use in the other two case studies), see Fig. 5a. Consequently, in Târnave, both the share of vineyards with high abundance of *L. botrana* and spider diversity significantly increased due to policy 5-NI (Fig. 5f, g). Policy 6-PO significantly increased pheromone dispenser use in Leithaberg and Târnave (Fig. 5b) and consequently reduced the share of vineyards in Leithaberg with high *L. botrana* abundance (Fig. 5f). This policy (6-PO) did not affect *L. botrana* abundance in Târnave due to the frequent use of insecticides there. Policy 7-FR significantly decreased the use of synthetic fungicides in all case studies and the use of copper- or sulfur-based fungicides in Palatinate (Fig. 5c, d). Lastly, the only significant change of *L. botrana* pupae predation rate was found by policy 4-CA in Palatinate, where this new policy increased predation (Fig. 5e): note this ecological process is affected by both fungicide use and inter-row vegetation management.

Besides the immediate effects of implemented polices on their respective behaviours, we also found some statistically significant indirect effects. These indirect effects highlight winegrowers’ adaptation to a policy beyond its original target. For example, in Leithaberg, the policy 2-CS (mandatory vegetation cover on steep vineyards) not only had expected effects on vegetation cover and consequent soil loss control, but also led to a statistically significant increase in the use of pheromone dispensers (Fig. 5b and Appendix 4) and consequently lower share of vineyards with high *Lobesia* abundance (Fig. 5f and Appendix 4). This indirect effect can be explained as follows: in the modelled system, the mandated vegetation cover can reduce yield potential, for some winegrowers this reduction became a concern and they responded to the problem by identifying a strategy to compensate yield potential loss, which was to adopt pheromone dispensers if it was not installed in their vineyards. Installing pheromone dispensers increased yield potential and decreased the pest abundance of *Lobesia* in their vineyards. Another example was found in Palatinate, where the policy 6-PO (pheromone dispenser obligation) did not affect the pheromone use situation (was already well implemented there) but instead led to a statistically significant decrease in use of seed mixture as the vegetation type in their inter-rows (Fig. 4c and Appendix 4) and consequently statistically significant increase in use of spontaneous vegetation (not shown in results, can be derived by 1 minus the value on Fig. 4c) as the alternative vegetation type. This could be related to our input ecological relationships which showed that higher pest predation rate in vineyards inter-rows with spontaneous vegetation compared to seed mixture use. However, as seed mixtures are comprised of more diverse plants than spontaneous vegetation, plant diversity was consequently reduced (Fig. 4f and Appendix 4).

## Discussion and Conclusions

### Implications for Policy-Making to Promote Ecosystem Services and Biodiversity in Viticulture

European viticultural landscapes need to be greener and reduce pesticide use (Chen et al. 2022; Paredes et al. 2021; Pertot et al. 2017; Winter et al. 2018), which requires behavioural change of winegrowers. Greener vineyards mean that bare soil management must be limited, and ideally inter-rows should be covered with diverse vegetation. Such behavioural change is expected to lead to improvements in several ecosystem services such as soil erosion control, biological control of vineyard pests, landscape aesthetics, and biodiversity in these landscapes, making them more environmentally friendly while providing a sustainable, less input-dependent form of agricultural management.

Policies can foster behavioural changes towards more sustainable management practices supporting biodiversity conservation and ecosystem service provision. Here, we assumed 100% implementation. Still the policy impacts vary between case study regions. For example, in the case of Târnave (RO), all policies aiming at increasing inter-row vegetation cover (2-CS, 3-CW, 4-CA) increased the share of vineyards with vegetation in every inter-row (Fig. 4a) and decreased the share of bare soil vineyards (Fig. 4b). Only two policies (2-CS and 4-CA) resulted in substantial and significant decrease of soil loss in this region (Fig. 4e). The same policies (2-CS, 3-CW, 4-CA) also increased the share of vineyards with vegetation in every inter-row and hence decreased soil erosion for Palatinate (DE) and Leithaberg (AT). However, the increase in share of vegetation cover and the decrease in soil loss in these two cases due to the vegetation related policies were less profound as compared to those in Târnave (RO), since in Palatinate (DE) and Leithaberg (AT) inter-row vegetation cover is much more common there at present. This is most likely related to the long-term implementation of agri-environmental policies supporting inter-row vegetation cover to combat soil erosion (e.g. OEPUL programme in AT). In a similar vein, a policy of banning insecticide use to control the pest *L. botrana* (policy 5-NI) does not make a difference for Palatinate and Leithaberg where these insecticides are currently not used due to the widespread use of pheromone dispensers against *L. botrana*. However, the impact of policy 5-NI in Târnave was substantial, leading to increasing pest abundance (Fig. 5f) but also an increase in spider diversity (Fig. 5g). Furthermore, small but statistically significant increases were also found under policies addressing inter-row vegetation cover (2-CS, 3-CW, and 4-CA) for spider species richness, suggesting that higher vegetation cover and related higher plant species richness also benefits spiders in vineyards (Blaise et al. 2022). In addition, these results underline the importance of the interconnected ecological relationships implemented in our ABM (Fig. 2) and the possibility of amplified effects when multiple policies are in order: for example, when both inter-row vegetation and insecticide ban are implemented. However, a policy banning insecticide use needs to be coupled with compensation payments supporting the implementation of pheromone dispensers against *L. botrana* at the landscape scale. Due to the scope of this study, possible synergistic effects of multiple policies are not explored in this study. This is an avenue for further investigation.

These modelling results strongly indicate the need to adapt policy instruments to the local context—as the current status and potentials concerning a target of an Agri-Environmental Programme (AEP) and the choice of measures vary greatly between case study regions. The principle of subsidiarity of the European Union can be seen as a case where decisions are supposedly taken at the institutional level that is best suited. However, the freedom of the respective member states for designing national AEPs, rules and regulations is also a drawback, considering that there is currently a lack of policies supporting vegetation cover in vineyard inter-rows or lower pesticide use in Romania, where there would be a great potential for improving soil erosion mitigation, other ecosystem services and biodiversity conservation. The comparably low uptake of AEPs per utilised agricultural area (UAA) of 11.1% in Romania is probably related to the focus on rural development and the transformation of governance systems there (Vesterager et al. 2016). Therefore, when national-level objectives and priorities differ from a common EU-level objective it becomes unlikely that national-level AEP can realise the (preservation) potential. Furthermore, the obligatory co-financing of agri-environmental programmes most probably also reduced their implementation (Vadineanu et al. 2013). In contrast, the uptake of AEPs per UAA of 69% in Austria is much higher than the EC 27 level of 22.2 % (Vesterager et al. 2016), which is also reflected in the higher share of vineyards with vegetation cover. As Romania is one of the newest EU member states, it could probably take more time to implement AEPs at a larger scale. In addition, reducing the required co-financing for AEPs (so that the uptake in Romania is not dependent on national contribution given their current budget limitation and political focus) and increasing environmental awareness of the population could also be a future driver of policy change there.

The indirect effects of policies found in our model results have a few implications. It highlights that winegrowers not only respond to a policy itself (the literal requirement from the policy) but also adapt other behaviours as they (1) have a different objective than what is targeted by the policy and/or (2) have multiple objectives. As a result, a policy can have effects beyond its original target—unforeseen or even unintended. The first example found in our model results shows that a vegetation cover policy also led to more uptake of an alternative of insecticide use in one case study; while the second example shows that a pesticide-related policy led to a reduction in plant diversity due to changes in inter-row vegetation type. It therefore becomes crucial for future models to identify if a policy can have undesired and unintended consequences. For example, in Austria, some farmers removed semi-natural elements (trees, shrubs, hedges, etc.) before their existence was made known to the authority, which was planning to implement a mandatory preservation of these elements.

Finally, these modelling results hint at efficiency of different policies and could inspire a discussion on priority setting given limited budget. For example, all of the inter-row vegetation policies (2-CS, 3-CW, and 4-CA) increase the share of vineyards with vegetation in every inter-row (Fig. 5a) and decrease the share of bare soil only vineyards (Fig. 5b). The policy targeting at all vineyards (4-CA) performs the best, and the policy targeting at vineyards on steep slopes (2-CS) performs better than the policy targeting at vineyards with water problems (3-CW). Nevertheless, only the first two policies result in substantial and significant decrease of soil loss in this region (Fig. 5e). This means that if targeting at all vineyards is a too costly intervention, efforts should be directed to those on steep slopes.

### Implications for Modelling Coupled Socio-Ecological Systems: Advances, Limitations, Challenges

Schlueter et al. (2017) named four research needs for modelling socio-ecological systems: (1) going beyond rather simple specifications of human decision-making, (2) developing strategies to deal with (irreducible) uncertainties, (3) more explicit modelling of feedbacks between the social and ecological systems, and (4) a conceptual and methodological framework for analysing and modelling socio-ecological systems. With our contribution, we focussed on the first and third research needs identified by Schlueter et al. (2017).

Current models on viticulture mostly simulate behaviours that are typical in other agricultural systems such as land use change (binary decision between growing grapevines vs. another crop) and adoption of sustainable practices (binary decision between conventional and organic farming) (Delay et al. 2015, 2017; Lammoglia et al. 2019; Martin-Clouaire et al. 2016; Zottele and Delay 2014). Here, we zoom in to simulate specific viticultural practices by using inputs from an empirical study (Chen et al. 2022). Many approaches exist to parameterise human agents in an empirical ABM (Smajgl and Barreteau 2017). To develop our model suite, we combined a survey with interviews since there was no consistent data on winegrowers’ actual behaviour available. Whereas the survey (Chen et al. 2022) informed primarily the initialisation of the model including winegrowers’ initial behaviours based on machine learning-generated decision-trees, the interviews and open questions in the survey informed adaptations of behaviours to climate change or policies. Combining a large survey dataset and machine learning is a powerful tool, however, it is limited to past decisions and behaviours (observed from the datasets) since changing environmental conditions in turn change the context for future decisions (An 2012). Responses to completely new policy instruments or other new developments, such as to plant fungus-resistant grapevine varieties that significantly reduce the required fungicide use, cannot be captured with such an approach. Therefore, future research incorporating empirically-based ABMs should look closely into how to inform decision-making when the context of decision-making differs from past contexts in a significant way.

Current viticultural models rarely couple behaviour with ecological processes (Delay et al. 2015, 2017, Elsawah et al. 2015; Lammoglia et al. 2019; Martin-Clouaire et al. 2016; Zottele and Delay 2014) with the exceptional model of Tissot et al. (2019). Here, we incorporated not only the ecological consequences of winegrowers’ behaviour in the model, but also feedbacks from the ecological subsystem to the winegrowers via losses of yields, soil or biodiversity. Due to these feedbacks, a policy instrument may lead to further adaptation from winegrowers beyond what the policy prescribes. Such indirect effects on the behaviours as well as their ecological impacts would be overlooked if the feedbacks were not included in the model. Such feedbacks may in the future also include local interactions such as neighbourhood effects. Since the model represents vineyards and the local environment in a spatially explicit way, such effects can be easily incorporated based on new empirical evidence in the future.

We looked at three case studies in parallel. Already during the design phase, we put emphasis on which processes and parameters are case-study specific and which ones are not. Differences not only exist in decision-making of winegrowers but also in how ecological processes work in detail for different biogeographical contexts (Beaumelle et al. 2021; Biddoccu et al. 2020; Gregorich 2020; Hall et al. 2020; Louis et al. 2002; Paredes et al. 2021; Reiff et al. 2021; Schirra and Louis 2001; Sharley et al. 2008). As an example, we found a positive impact of organic farming on natural pest control in vineyards of Bordeaux (FR) but a negative effect in Leithaberg (AT), which is most likely related to the different pesticide associated with the respective farming systems in those different wine-growing (Beaumelle et al. 2021; Möth et al. 2021; Reiff et al. 2021). In addition, different natural enemies react differently to pest and inter-row management which is reflected in the overall decrease of predatory mites in organic vineyards in both Bordeaux and Leithaberg (Möth et al. 2023). This experience further highlights the challenge to apply one empirically-based model to another context.

We most likely underestimated the variability in ecological processes across case studies. When selecting the three case studies, we focussed on data availability for modelling winegrowers’ decision-making. The model suite was originally planned for two additional case study regions (i.e., Montilla-Moriles in Spain and Bordeaux in France). However, survey responses from these two regions were not sufficient (10 from Montilla-Moriles and 15 from Bordeaux) to inform modelling which allows for case-specific explorations (Sun et al. 2016). Thus, the three modelled case study regions are a convenience sample based on socio-economic data. For the ecological processes, we were partly able to draw on field work conducted by colleagues in our project and other literature. However, in many cases, we could only draw on relationships published for other cases (e.g. relation between insecticide use and *L. botrana* abundance, based on Paredes et al. 2021 for Spanish vineyards), without being able to verify whether these relationships actually vary between our case studies. For the design on climate change, we only implemented one scenario, and our knowledge and confidence on how exactly temperature (number of warmer and a lot warmer years) and precipitation (the number of drier, wetter, and normal years) will be distributed over the simulated period is very limited. Future study that looks more specifically into the effect of climate change should systematically vary the design to investigate if and how model outcomes on behaviours as well as ecological impacts would differ.

Validating such a complex model is a challenging task (An et al. 2021, Troost et al. 2023). Here, we focused on comparing model output on winegrowers’ behaviours with estimates provided by local experts. Only one or two local experts per case study provided their input and agreed upon one joint opinion before providing their feedback to us. While this approach spared us the need to deal with contradicting statements, experts reaching a consensus can lead to suppression of opposing views and a tendency towards the mean. Many of the ecological indicators would require extra data collection in the field to provide estimates, so we decided to skip validating that part and only indicated results that we found questionable in Appendix 3. For both parts, collecting more data and harmonising reporting schemes on agricultural practises would have been beneficial. Since the estimates by local experts were not complete and only based on one or two opinions, we refrained from conducting a quantitative output validation (methods summarised in Troost et al. 2023).

Finally, challenges remain when policies are explicitly represented in ABMs of socio-ecological systems. To explore the potential effects of considered policy scenarios, we assumed all policies are implemented in reality and in a similar way. However, policies are known to be implemented differently, e.g., with or without controls that deal with rule-breaking and enforcement cost (Alston and Gaeta 2021). This is an avenue for future research with a focus on political science.

### Supplementary Information

Appendix

Appendix

Appendix

Appendix
