# Supplementary material for: Exploring Winegrowers’ Behaviours and Ecological Impacts Under Climate Change and Policy Scenarios—Examples from Three European Winegrowing Regions
Source: Environ Manage. 2024 Jan 11;73(4):841–57. doi: 10.1007/s00267-023-01924-8 (PMC10973083; doi:10.1007/s00267-023-01924-8)
Supplement: Supplementary file 1 — Appendix [file 267_2023_1924_MOESM1_ESM.docx]

**Exploring Pest and Inter-row management in European Vineyards and their potential Impacts (EPIEVI)**

**Link:** [**https://www.comses.net/codebases/7922d774-be9c-4719-9342-8f5d4db866c6/releases/1.0.0/**](https://www.comses.net/codebases/7922d774-be9c-4719-9342-8f5d4db866c6/releases/1.0.0/)

**Model description**

This model description document follows the ODD+D standards and suggestions from Müller et al. (2013) for describing human decisions in agent-based models.

I) **Overview**

I.i Purpose

I.i.a What is the purpose of the study?

The purpose of this study is to explore the potential impacts of pest and inter-row management of European winegrowers in response to policy and climate change scenarios. **Pest management** considered in this study include insecticides, pheromone dispensers (as an alternative to insecticides), fungicides (both the synthetic active ingredients and copper- and/or sulphur-based). **Inter-row management** concerns the arrangement of vegetation (every row, every 2^nd^ row, or only bare soil) in the inter-rows and the type of vegetation (spontaneous vegetation or seed mixture). **Winegrowers** are considered as European winegrowers in general and winegrowers from three regions (i.e., Leithaberg in Austria, Palatinate in Germany, and Târnave in Romania). **The ecological impacts** of winegrowers’ behaviours include grapevine yield potentials, soil erosion, pest abundance level (*Lobesia botrana* (Denis & Schiffermüller)), pest control (predation of *L. botrana* pupae), biodiversity (plant and spider), and landscape aesthetics. **Policy scenarios** include the consideration of mandatory vegetation cover and obligation to reduce pesticide. **Climate change** scenario concerns the trend of warmer but oscillating precipitation patterns.

**I.i.b For whom is the model designed?**

The model is designed for scientists and policymakers but also as a tool to communicate and discuss model results with other important stakeholders such as winegrowers and experts from extension services.

I.ii Entities, state variables and scales

I.ii.a What kinds of entities are in the model?

The main entities include: winegrowers (land users, represented by agents, i.e. “turtles” in NetLogo) and their vineyard plots (land parcel, represented by cells, i.e. “patches” in NetLogo). These entities have dynamic attributes and interactions.

There are also static entities in the model, i.e. the land parcels representing urban settlements, other agriculture, forest and semi-nature, and water. They together make up the environment of the landscape.

I.ii.b By what attributes (i.e. state variables and parameters) are these entities characterised?

Winegrowers are characterised by their personal characteristics (including age, gender, experience in viticulture, education), their attitudes (in four groups of economic, social, environmental, and adaptive capacity), and management characteristics (including vineyard size, farming type, number of varieties cultivated, producing for a label or not, subsidy status, offering tourism activities or not) (Table 1 and Table 2).

Winegrowers and their vineyard plots have a 1-to-many relationship. Each winegrower has a set of vineyard plots, which make up his/her vineyard. Each vineyard, as represented in the model world, mostly take the shape of long rectangles, just as it is seen in the real world.

Patches only have the id of their winegrower as an attribute.

**Table 1. State variables (that can change during a model run) of agents**

| Variable | Description | Domain |
| --- | --- | --- |
| IR-management | Arrangement of inter-row vegetation | "every row", "every 2nd", or "bare soil" |
| IR-type | The type of inter-row vegetation | "no vegetation", "seed mixture", "spontaneous" |
| Insecticide | The annual spraying frequency of insecticide | # (per year) |
| Pheromone | Whether or not the agent is using pheromone dispensers | True, false |
| Synthetic-fungicide | The annual spraying frequency of synthetic fungicides | # (per year) |
| Organic-fungicide | The annual spraying frequency of copper- and/or sulphur-based fungicides | # (per year) |
| my-soil-loss | Annual soil loss in my vineyard | ton/ha |
| my-abundance-lobesia | The abundance level of the pest *L. botrana* | “none”, “low”, “medium”, “high” |
| my-pupae-predation | The predation rate of the pupae of the pest *L. botrana* | %, dimensionless |
| my-spider-diversity | Diversity of spiders from my vineyard at the species level | # (count of species) |
| my-plant-diversity | Diversity of vascular plant from the inter-rows in my vineyard at the species level | # (count of species) |
| my-potential-yield-up | Number of times when the yield potential is increased | # (count of events that lead to increase of yield potential) |
| my-potential-yield-down | Number of times when the yield potential is decreased | # (count of events that lead to decrease of yield |
| yield-problem | If yield is considered as a problem at the vineyard level | True, false |
| soil-problem | If soil loss is considered as a problem at the vineyard level | True, false |
| biodiversity-problem | If biodiversity is considered as a problem at the vineyard level | True, false |
| priority | The problem chosen as the priority to solve, if more than one | "soil", "yield", or "biodiversity" |
| cause-by-fungicide | Whether the problem is caused by fungicide use | True, false |
| cause-by-insecticide | Whether the problem is caused by insecticide use | True, false |
| need-pheromone | Whether there is a need to use pheromone dispenser | True, false |
| cause-by-IR | Whether the problem is caused by inter-row management | True, false |
| intervention | An action identified by the winegrower to tackle the problem | "Synthetic fungicide +/-", "Organic fungicide +/-", "Insecticide +/-", "IR +/-", "Pheromone +" |
| obligation | An obligation for the winegrower due to the introduced policy | “cover”, “no insecticide”, “pheromone”, “fungicide reduction” |
| adaptation | An adaptation identified by the winegrower due to climate change | "less fungicide and insecticide", "less fungicide", "more fungicide and insecticide", "more fungicide" |

**Table 2. Parameters (that do not change during a model run) of agents**

| Parameter | Description | Domain |
| --- | --- | --- |
| location | Spatial position in the map of an abstracted viticultural landscape | GIS coordinates |
| vineyard-size | Vineyard size operated by the winegrower | hectare |
| mean-slope | Average slope of vineyard plots, owned/managed by this winegrower | %, percentage rise |
| management-type | The management type of the vineyard according to standard agricultural practices | "Biological", "Integrated", "Conventional", or "In transition to bio." |
| n-varieties | The number of varieties grown in the vineyard | # |
| lack-water? | Whether or not the vineyard is affected by water shortage | True, false |
| tourism? | Whether or not tourism activities are offered at the vineyard | True, false |
| label? | Whether or not the winegrower produces for a label. | True, false |
| all attitude related attributes^^[[1]](#footnote-1)^^ | The extent to which the attitude is perceived by the winegrower as important in relation to his/her viticultural practices | [1, 5] with 1 being not important at all, and 5 being very important. |

**I.ii.c What are the exogenous factors/drivers of the model?**

The exogenous drivers of the model include: policy and climate change scenario. The considered policy scenarios are: mandatory vegetation cover in the inter-rows for vineyards on slopes, mandatory vegetation cover in the inter-rows for vineyards without water shortage problem, mandatory vegetation cover for all vineyards, insecticide ban, mandatory pheromone dispenser use, and mandatory fungicide reduction. The considered climate change scenario describes a warmer future for European viticultural landscape but oscillating precipitation, compared to the current situation.

**I.ii.d If applicable, how is space included in the model?**

Space is explicitly represented in the model. For each case study (Leithaberg, Palatinate, and Târnave, as well as a generic European viticultural landscape), a 10 km x 10 km landscape from the corresponding region is imported to (or generated in) the model, based on the Corine Land Cover Map (2018). These landscapes contain the land use/cover types of urban settlements, viticulture, other agriculture, forest and semi-natural elements, and wetlands and water. The share of viticultural area in each landscape is similar (26-27 %). In order to avoid identification of specific locations and undesired personal attachment to these locations from winegrowers to whom the model is presented, we abstract each landscape so that the share of different land use/cover roughly remains but the shape of these elements may be varied and simplified (Figure 1).


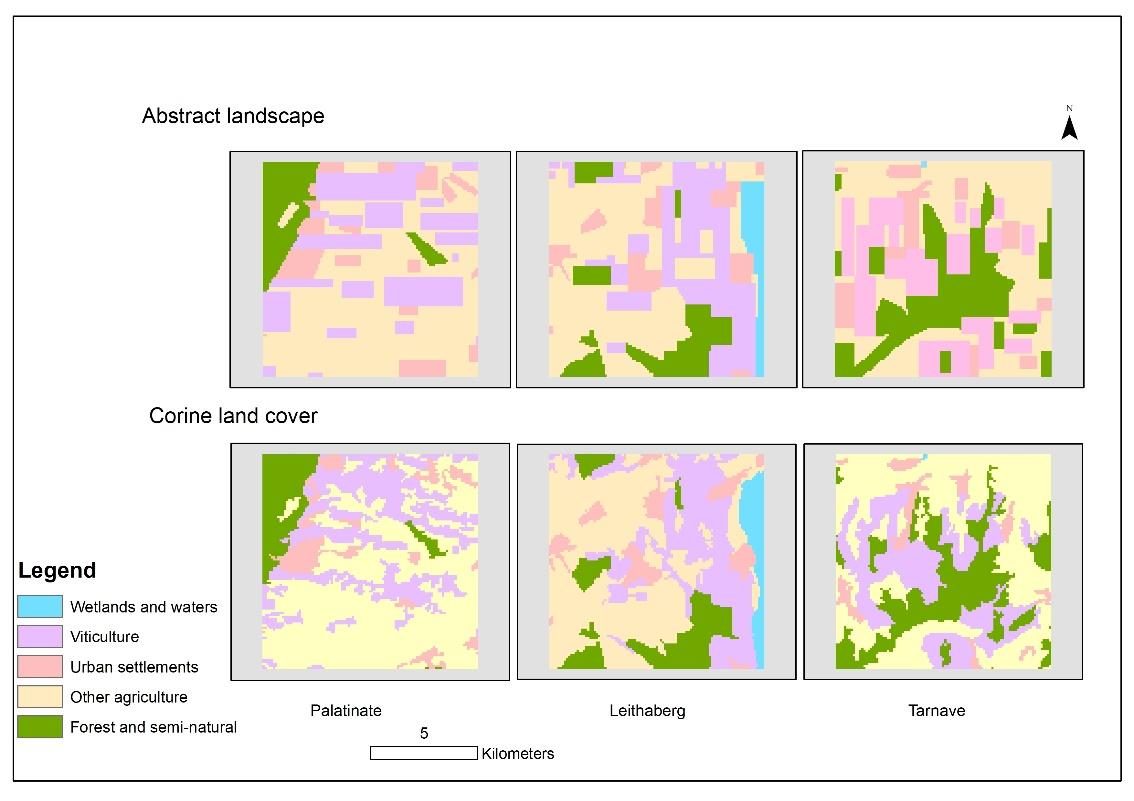


**Figure 1. From actual land cover to abstract landscape in each case study for the agent-based model spatial configuration**

I.ii.e What are the temporal and spatial resolutions and extents of the model?

Temporal: resolution: 1 year; extent: 10 years for the simulations to investigate the response of the modelled viticultural system to policy scenarios. In practice the model can be run for longer period of time without considering climate change (or with, if a longer timespan of climate conditions is specified).

Spatial: resolution: 100 m; extent: 10 km x 10 km.

**I.iii Process overview and scheduling**

**I.iii.a What entity does what, and in what order?**

Each time step starts with agents implementing any planned behavioural changes concerning inter-row and pest management, either as self-identified intervention to existing problems or as fulfilment of their obligation due to an introduced policy (if any) from the previous step. In the very first time step, agents will repeat their initial behaviours. All following time steps start with agents either continuing their behaviour from previous time steps or implementing behavioural changes they decided upon in the previous step. After implementing their behaviour, agents check if adaptation to the current climate conditions is needed which may affect their pest and fungal disease control. With all planned behavioural changes and adaptations implemented, these behaviours are updated and recorded. The ecological impacts of these behaviours are then determined by the ecological sub-models. Then agents check if a new policy is introduced and if they are affected by the policy and prepare for a behavioural change in the following time step. In the end of each time step, agents determine if there are any problems concerning yield, soil, and biodiversity. If the problem becomes a concern, they identify and cause of the problem (in relation to their inter-row and pest management) and decide upon how to change their behaviour in the following time step.

The pseudocode of the main simulation cycle is as follows. Sub-models (in bold) are further explained in section III.iv.

Initialisation {

set-seed-setup ;set the random seed for the model initialisation

initialise-map ;import or generate the case-study land use and cover map

show-land-use ;visualise land use and cover for the case-study

initialise-winegrowers ;generate winegrowers

allocate-vineyards ;allocate the winegrowers to their vineyards

initialise-attributes ;generate winegrower and vineyard attributes

**initialise-behaviours** ;initialise winegrowers behaviours based on empirical evidence

behaviour-control ;check the initialised behaviours

remember-initial-behaviour ;record the initial behaviours

initialise-globals ;initialise global variables for the model

report-this-setup ;report key information of this setup in the interface

**report-ecological** ;link the initial behaviour with their ecological impacts

}

For each time step {

**implement-behavioural-change**

**check-weather-and-adaptation**

**update-behaviour-status**

**update-ecologicals**

**check-policy**

**check-problems**

**check-causes**

**check-options**

}

II) **Design Concepts**

**II.i Theoretical and Empirical Background**

**II.i.a Which general concepts, theories or hypotheses are underlying the model's design at the system level or at the level(s) of the submodel(s) (apart from the decision model)? What is the link to complexity and the purpose of the model?**

The concept of social-ecological systems (Ostrom, 2009; Schlueter et al., 2012), or Coupled Human and Natural systems (Liu et al., 2007) are underlying the model’s design at the system level: on the one hand, winegrowers as agents decide on how they manage their inter-rows and use various pesticides; on the other hand, these behaviours have ecological consequences concerning yield potentials, soil loss, pest abundance, pest control, biodiversity, and landscape aesthetics. These ecological impacts will feed back to affect winegrowers’ inter-row and pest management. The model addresses the following issues in relation to complexity: 1) an explicit representation of **heterogeneity** in both the attributes of agents and their behaviours, 2) an explicit representation of the **coupling** and hence **the feedback** between the process of human decision making and various ecological processes. As a result, these considerations allow the model to simulate the change of winegrowers’ behaviours and the ecological consequences of these behaviours.

**II.i.b On what assumptions is/are the agents' decision model(s) based?**

For the initialisation of the agents’ behaviour, as well as the change of agents’ behaviour, the rules are based on empirical evidences (Chen et al., 2022) in the form of decision trees.

The following assumptions are made in the sub-model to identify problems: agents who have high economic attitudes will respond to decrease of grapevine yield potential; agents who have high soil preserving attitude will respond to increase in soil loss; and agents who have high biodiversity preserving attitude will respond to decrease in biodiversity.

The following assumptions are made in the sub-model to prioritise: agents who have multiple problems (yield decrease, soil loss increase, biodiversity decrease) will randomly choose one of the problems to deliberate the cause and intervention as a solution to this problem for the next time step. This is also validated by interviewing winegrowers.

The following assumption is made in the sub-model to respond to policy: no rule breakers are modelled.

**II.i.c Why is/are certain decision model(s) chosen?**

All decision models are based on empirical evidences.

**II.i.d If the model/submodel (e.g. the decision model) is based on empirical data, where do the data come from?**

Empirical data of winegrowers’ inter-row and pest management came from the collected questionnaire survey between 2019 and 2020 (Chen et al., 2022). Besides, more data (see Table 3) is collected for various purposes.

**Table 3. Collected data for the ABM**

| **Collection method** | **Collection time** | **Purpose of data collection** |
| --- | --- | --- |
| Questionnaire survey distributed to winegrowers in five European viticultural regions | Between 2019 and 2020 | To initialise agent behaviours in this model |
| Focus group discussions and interviews with winegrowers | Focus group discussion (2019-2020)  Interviews 2021 | To verify the decision process in the model |
| Literature review and data analyses on ecological impacts of various behaviours | 2021-2022 | To provide parameters to the model in order to link human behaviours with their ecological impacts |
| Regional level statistics related to viticultural practices provided by local experts | 2021-2022 | To validate the model results |

**II.i.e At which level of aggregation were the data available?**

The data were available at the individual winegrower level, which corresponded to the agents in this model.

**II.ii Individual Decision-Making**

**II.ii.a What are the subjects and objects of the decision-making? On which level of aggregation is decision-making modelled? Are multiple levels of decision making included**?

Subjects: individual winegrowers;

Objects: vegetation cover for the inter-rows in the vineyards (whether or not and the type of), and frequency of spraying of insecticides (and alternative of pheromone dispensers) and fungicides;

Level of aggregation of the decision-making: vineyard level, the whole vineyard (including all land parcels) managed by this winegrower. All decisions (in relation to inter-row and pest management) are at the level of the vineyard.

**II.ii.b What is the basic rationality behind agent decision-making in the model?** Do agents pursue an explicit objective or have other success criteria?

The basic rationality behind agent decision-making is to sustain viticulture. Depending on their attitudes, agents pursue explicit objectives to secure grapevine production (yield), to control soil erosion, and to maintain biodiversity.

**II.ii.c How do agents make their decisions?**

Agents are initialised with their habitual behaviours concerning inter-row and pest management under normal circumstances. They repeat such habitual behaviours as long as 1) there are no problems with their grapevine yield potentials, soil erosion, and biodiversity; 2) no need to adapt to climate and weather conditions in the current time step; and 3) no newly introduced policies which requires a change on their current behaviour. A problem is defined as the change of yield, soil, or biodiversity larger than the tolerance threshold of the agent (see section III.iv.b). Once an agent identifies a problem, (s)he deliberates the cause and a solution of the problem, in relation to inter-row and pest management. The solution will be implemented in the next time step. The adaptation to climate conditions and policy scenarios will be described in the next section.

**II.ii.d Do the agents adapt their behaviour to changing endogenous and exogenous state variables? And if yes, how?**

Agents adapt their behaviour to both endogenous and exogenous state variables.

Endogenously, agents have three state variables called “yield-problem”, “soil-problem”, and “biodiversity-problem”. These variables can change their states to true, which then trigger agents to find the cause and solution of the problem, with regards to inter-row and pest management.

Exogenously, the climate scenario used in the model per time step may change the state variable of winegrowers called “weather-problem” to change the states to true, based on collected empirical evidence. Agents will adapt to the current climate conditions (temperature and precipitation) by adjusting their pesticide use, based on collected empirical evidence.

Another exogenously determined state variable is called “obligation”, this is controlled by externally designed policy interventions regarding inter-row and pest management. Agents whose “obligation” are changed due to the newly introduced policy will respond to the content of the policy accordingly.

**II.ii.e Do social norms or cultural values play a role in the decision-making process?**

The identification of problems, the causes and solutions are implemented at individual level by taking into account various agent attributes. However, when agents decide to grow vegetation in the inter-rows of their vineyards where inter-rows used to be bare soil, they would use the type of vegetation that is mostly chosen by other winegrowers.

**II.ii.f Do spatial aspects play a role in the decision process?**

The explicit spatial representations of different land use/cover types in the landscape as well as the shape of the vineyards are model design choices in order to use such visual representation to communicate with winegrowers and other stakeholders. For decision process, slope is a relevant factor that affect winegrowers’ decision and the ecological process of soil erosion.

**II.ii.g Do temporal aspects play a role in the decision process?**

Agents’ behaviour at time step t leads to certain ecological impacts, which over time can feed back to change their behaviour at time step t + n.

**II.ii.h To which extent and how is uncertainty included in the agents' decision rules?**

When an agent identifies multiple problems, they randomly choose one of them to solve at a time.

**II.iii Learning**

**II.iii.a Is individual learning included in the decision process? How do individuals change their decision rules over time as consequence of their experience?**

Individual learning is included in the decision process. For a given problem (e.g., decrease in yield potentials), which may be caused by several behaviours, an individual agent may identify a cause and the corresponding intervention for the next time step. If the problem remains, the agent may identify another cause and intervention.

**II.iii.b Is collective learning implemented in the model?**

Collective learning is not implemented in the model.

**II.iv Individual Sensing**

Individual agents sense the changes of 1) yield potentials, 2) soil loss, 3) biodiversity in their own vineyards and they also sense 4) climate conditions, and 5) newly introduced policies that may affect their behaviour and 6) the vegetation type of other vineyards.

**II.v Individual Prediction**

Based on empirical evidence, winegrowers deal with inter-row and pest management in an adaptive manner. Therefore, no predictions are included.

**II.vi Interaction**

Given the collected empirical evidence suggesting no direct interactions between the winegrowers, no interaction is implemented in their decision process. Indirectly, the mostly used vegetation cover type can be observed and used by an agent who decides to change from bare soil to vegetated inter-rows.

**II.vii Collectives**

No collectives are modelled. The collected empirical evidence does not suggest collectives.

**II.viii Heterogeneity**

**II.viii.a Are the agents heterogeneous? If yes, which state variables and/or processes differ between the agents?**

Agents are heterogeneous. All of the state variables (that may change over time in the model) in **Table 1** and the attributes (that do not change over time in the model) **Table 2** can differ between agents. The heterogeneity is based on empirical evidence.

**II.viii.b Are the agents heterogeneous in their decision-making? If yes, which decision models or decision objects differ between the agents?**

Initial behaviours of agents represent the habitual behaviour that agents would repeat over time, unless situations change (see II.ii.c). These initial behaviours differ across contexts, based on empirical evidence. As the case study change from one region to another, the rules to initialise the behaviours change as well. These rules are derived from the empirical study. Also, the adaptation to climate conditions also differs across the case studies, based on empirical evidence.

**II.ix Stochasticity**

II.ix.a What processes (including initialisation) are modelled by assuming they are random or partly random?

For initialisation, the viticultural region of the imported/generated landscape is randomly partitioned to create long rectangles of different sizes that are operated by different agents. The initial behaviours of the agents are based on the rules derived from empirical study, in which randomness exists: for example, for all agents who meet certain criteria on their attributes, there is x% chance to find that they have vegetation in every inter-row and y% chance to find vegetation only in every 2^nd^ inter-row, and (1-x-y)% change to find no vegetation. These values are taken from the empirical study, which yield the minimum error.

For the simulation process, randomness exists in the order of agents taking actions and also in their problem solving: when an agent is confronted with more than one problem, (s)he will randomly choose one problem, be it yield, soil, or biodiversity, to identify the cause of the problem and an intervention of the problem as solution.

**II.x Observation**

II.x.a What data are collected from the ABM for testing, understanding and analysing it, and how and when are they collected?

Both the statistics of agents’ behaviour and their ecological impacts are collected from the ABM by the end of each simulation. See Table 4 for details.

**Table 4. Model output measures**

| Agents’ behaviour | Share of winegrowers with bare soil only in the inter-rows | Statistics |
| --- | --- | --- |
|  | Share of winegrowers with vegetation in every 2^nd^ inter-row | Mean of all time steps |
|  | Share of winegrowers with vegetation in every inter-row | Mean of all time steps |
|  | Share of winegrowers without insecticide use | Mean of all time steps |
|  | Share of winegrowers using pheromone dispensers | Mean of all time steps |
|  | Mean annual application of insecticide | Mean of all time steps |
|  | Mean annual application of synthetic fungicide | Mean of all time steps |
|  | Mean annual application of copper-and/or sulphur-based fungicide | Mean of all time steps |
| Ecological impacts | Mean annual soil loss of vineyards | Mean of all time steps |
|  | Mean plant diversity of vineyards | Mean of all time steps |
|  | Mean spider diversity of vineyards | Mean of all time steps |
|  | Mean predation rate of *Lobesia botrana* pupae on vineyards | Mean of all time steps |
|  | Share of vineyards with high *L. botrana* abundance | Mean of all time steps |
|  | Mean extent of vegetated inter-rows of vineyards | Mean of all time steps |
|  | Total times yield potentials are decreased (per vineyard) | Total (at the end of the simulation) |
|  | Total times yield potentials are increased (per vineyard) | Total (at the end of the simulation) |

II.x.b What key results, outputs or characteristics of the model are emerging from the individuals? (Emergence)

At the landscape (regional) level, several characteristics emerge from the behaviour of the individuals, they are:

- Behaviour statistics at this level, including inter-row management and the type of inter-row vegetation, the use of insecticides (yes or no, and annual frequency) and the alternative of pheromone dispensers (yes or no), and the use (annual frequency) of synthetic fungicides and copper- and/or sulphur-based fungicides;
- Landscape aesthetics, measured by the extent to which vineyard inter-rows are vegetated;
- Total and average soil loss in the vineyards;
- Level of pest abundance in the vineyards, measured by the pest *L. botrana*;
- The average vascular plant biodiversity in the vineyards;
- The average spider biodiversity in the vineyards;
- The average pest control by natural predators in the vineyards, measured by the predation rate of the pupae of the pest *L. botrana*.

**III) Details**

**III.i Implementation Details**

**III.i.a How has the model been implemented?**

The model is implemented in Netlogo 6.2.

**III.i.b Is the model accessible, and if so where?**

The model is accessible at the CoMSES.

**III.ii Initialisation**

III.ii.a What is the initial state of the model world, i.e. at time *t* = 0 of a simulation run?

The initial state of the model world concerns both the winegrowers and their vineyards. For the winegrowers, their personal, attitude, and management properties are initialised in a way so that at the landscape level, their pest and inter-row management in their vineyards match with empirical observations. This initial state of the model world is considered as the current status of the viticultural landscape, which is ready to be simulated for future scenarios. To initialise the total landscape (contains other land cover/use) and the viticultural landscape (delineates the vineyards), an ascii file is needed for a specific case study and a generic landscape is generated randomly within the model if the case study is specified as “generic”. These ascii files for specific case studies are as follows:

- Leithaberg.asc
- Palatinate.asc
- Târnave.asc

III.ii.b Is the initialisation always the same, or is it allowed to vary among simulations?

The initialisation is allowed to vary among simulations.

III.ii.c Are the initial values chosen arbitrarily or based on data?

Initial values are based on data collected from a questionnaire survey. For the initialisation of the landscape for specific case studies (Leithaberg, Palatinate, and Târnave), we randomly took a 10*10 km landscape from this region based on the Corine land cover map and abstracted the landscape before they are imported as ascii files into Netlogo.

III.iii Input Data

III.iii.a Does the model use input from external sources such as data files or other models to represent processes that change over time?

The climate scenario is based on external sources. For simplicity, only one climate scenario is used. This scenario is defined by temperature and precipitation relative to the current conditions: is it warmer or colder, and is it drier or wetter. Based on literature (Ljungqvist et al., 2019) and climate monitoring of the relevant case studies (meteoblue, 2022b, 2022c, 2022a), a list of temperature change and a list of precipitation change, both relative to the current conditions, are used to define climate change for the next 10 years. The temperature list describes the future with years to be warmer and years to be a lot warmer and the precipitation list describes the future with wetter years, drier years, and normal years.

**III.iv Submodels**

III.iv.a What, in detail, are the submodels that represent the processes listed in ‘Process overview and scheduling’?

**Key submodels in initialisation:**

- **initialise-behaviours**

In this submodel, all relevant behaviours are initialised based on decision trees generated from questionnaire survey results. A total of 4 * 6 = 24 decision trees were used (4 case studies and 6 behaviours) to initialise behaviours. These decisions trees were found as the statistically best prediction on winegrowers’ behaviours.

- **report-ecological**

In this submodel, the initialised behaviours are connected to their ecological consequences concerning yield potential, landscape aesthetics, soil loss, pest abundance, pest predation, vascular plant diversity, and spider diversity. These quantitative relationships are based on parameter values in published articles (Biddoccu et al., 2020; Gregorich, 2020; Hall et al., 2020; Louis et al., 2002; Paredes et al., 2021; Reiff et al., 2021; Schirra & Louis, 2001; Sharley et al., 2008) and data analyses from natural scientists. These relationships are visualised in Figure 2: the squares are modelled behaviours and the ovals are the ecological variables, the links represent casual relationships, with quantitative values found in literature or data analyses implemented as model parameters.

There are six categories of ecological impacts resulting from agent behaviours:

**calculate-landscape-aesthetics**

Two global variables are calculated, based on winegrowers’ “IR-management” (if they have vegetation in every, or every 2^nd^ inter-row, or no vegetation but bare soil in all inter-rows), and “IR-type” (the type of the inter-row vegetation). First, “vegetated-interrows” is the share of vegetated inter-rows across the whole landscape; second, “native-interrows” is the share of the vegetated inter-rows where spontaneous vegetation grows.

**determine-soil-loss**

First, soil loss is calculated at each vineyard, based on “IR-management”, “mean-slope”, and the case study region. Parameters are derived from literature in which soil loss modelling was conducted for the relevant case study regions (Biddoccu et al., 2020), see Table 5. For each mean and standard deviation, an equivalent gamma distribution (alpha and lambda using a sample size of 200) was used in the ABM to generate soil erosion distribution for winegrowers in the specific category; then, we ordered each vineyard (winegrower) by mean-slope from high to low and assigned the generated soil erosion from largest to smallest to these vineyards/winegrowers. After the soil loss is calculated at the vineyard level (with the agent state variable “my-soil-loss”), a global variable “soil-loss” takes the mean across vineyards.

**Table 5. Determination of soil erosion by inter-row vegetation cover, based on (Biddoccu et al., 2020).**

|  | **Annual soil loss (ton/ha)** | | |
| --- | --- | --- | --- |
| **Case study** | **Bare soil only** | **Vegetation in every 2nd inter-row** | **Vegetation in every inter-row** |
| **Austria** | 21.2 (SD 15.29)  **α**:1.8 **λ**: 0.09 | 11.98 (SD 8.83)  **α**:1.54 **λ**: 0.13 | 3.31 (SD2.11)  **α**:2.63 **λ**: 0.8 |
| **Romania** | 22.24 (SD 11.13)  **α**:3.07 **λ**: 0.14 | 11.92 (SD 5.29)  **α**:5.92 **λ**: 0.5 | 1.75 (SD 1.36)  **α**:1.61 **λ**: 0.92 |
| **Germany & Generic** | 17.04 (SD 13.1)  **α**:1.66 **λ**: 0.1 | 10.71 (SD 7.35)  **α**:2.10 **λ**: 0.2 | 2.01 (SD 1.63)  **α**:1.73 **λ**: 0.86 |

**determine-*L. botrana*-abundance**

Based on literature (Louis et al., 2002; Paredes et al., 2021; Schirra & Louis, 2001), two behaviours are used to determine the level of *L. botrana* abundance: winegrowers’ use of pheromone dispensers and insecticides. Abundance level is “none” in vineyards with pheromone use and is “high” in vineyards with neither pheromone use nor insecticide use. The level is randomly assigned as “low” or “medium” in vineyards with only insecticide use. After *L. botrana* abundance is determined at the vineyard level, four global variables “lobesia-high”, “lobesia-medium”, “lobesia-low”, “lobesia-none” are calculated to account the number of vineyards with the corresponding pest abundance levels.

**determine-predation**

Based on literature (Reiff et al., 2021), two behaviours and one agent attribute - behaviours of “IR-type” and “fungicide”, and attribute of “management-type” (organic or conventional) - are used to determine the predation rate of *L. botrana* pupae at the vineyard level with the agent state variable “my-pupae-predation”. The quantities of the predation rate for each combination of viticultural practices are based on the published results (Reiff et al., 2021), see Table 6. For each mean and standard deviation, an equivalent gamma distribution (alpha and lambda using a sample size of 200) was used in the ABM to generate predation rate distribution for winegrowers in the specific category; then, we ordered each vineyard (winegrower) by total frequency of fungicide applications (synthetic + copper- and/or sulphur-based) from high to low and assigned the generated predation rate from smallest to largest to these vineyards/winegrowers. After predation rate is determined at vineyard level, a global variable “pupae-predation” takes the mean across vineyards.

**Table 6. Determination of *L. botrana*** **pupae predation rate, based on (Reiff et al., 2021).**

|  | ***L. botrana* pupae predation rate (%)** | |
| --- | --- | --- |
| **Type of vegetation in the inter-rows** | **Conventional** | **Organic** |
| **Spontaneous** | 93 (SD 4.2)  **α**:549.49 **λ**: 5.49 | 76.5 (SD 4.9)  **α**:231.31 **λ**: 3.02 |
| **Seed mixture** | 82.8 (SD 8.3)  **α**:103.41 **λ**: 1.25 | 76.1 (SD 9.7)  **α**:69.44 **λ**: 0.91 |

**determine-spider-diversity**

Based on data analyses, one behaviour and one agent attribute - “insecticide” and “management-type”- are used to determine the spider diversity (on grapevine foliage) at the vineyard level with the agent state variable “my-spider-diversity”. The data analysis shows that insecticide use decreases spider diversity while organic farming increases spider diversity. The quantities of spider diversity are based on results from this data analysis. As inter-row vegetation is also reported to affect spider diversity (Sharley et al., 2008), we implemented this relationship by reducing the diversity for vineyards with bare soil in the inter-rows by 1 and by adding 1 for vineyards with vegetation in the inter-rows.

**Table 7. Determination of spider diversity, based on data analysis and (Sharley et al., 2008)**

|  | **Spider diversity (Foliage)** | |
| --- | --- | --- |
| **Insecticide frequency (annual)** | **Conventional** | **Organic** |
| **0** | 7 | 8 |
| **1** | 6.25 | 7 |
| **2** | 5.5 | 6 |
| **>= 3** | 4.5 | 5.5 |

**determine-plant-diversity**

Based on literature (Hall et al., 2020), the behaviour “IR-type” and the case study region are used to determine the vascular plant diversity at the vineyard level with the agent state variable “my-plant-diversity”. The quantities of plant diversity are based on the published results, see Table 8. For each mean and standard deviation, an equivalent gamma distribution (alpha and lambda using a sample size of 200) was used in the ABM to generate plant diversity distribution for winegrowers in the specific category. After plant diversity at vineyard level is determined, a global variable “plant-diversity” takes the mean across vineyards.

**Table 8. Determination of plant diversity, based on (Hall et al., 2020)**

|  | **Plant diversity (vascular plant species)** | | |
| --- | --- | --- | --- |
| **Case study** | **Bare soil only** | **Seed mixture** | **Spontaneous vegetation** |
| **Austria** | 11.1 (SD 4.1)  **α**:6.78 **λ**: 0.61 | 15.9 (SD 4.1)  **α**:12.14 **λ**: 0.77 | 14.6 (SD 4.1)  **α**:12.27 **λ**: 0.84 |
| **Romania** | 11.9 (SD 4.1)  **α**:7.72 **λ**: 0.65 | 16.7 (SD 4.1)  **α**:16.02 **λ**: 0.96 | 15.4 (SD 4.1)  **α**:14.91 **λ**: 0.97 |
| **Germany** | 8.8 (SD 5.5)  **α**:2.39 **λ**: 0.27 | 19.9 (SD 4.32)  **α**:18.99 **λ**: 0.95 | 11.4 (SD 2.99)  **α**:15.39 **λ**: 1.35 |
| **Generic** | 8.8 (SD 5.5)  **α**:3.3 **λ**: 0.37 | 13.6 (SD 4.2)  **α**:8.41 **λ**: 0.62 | 12.3 (SD 5.5)  **α**:4.39 **λ**: 0.36 |

**
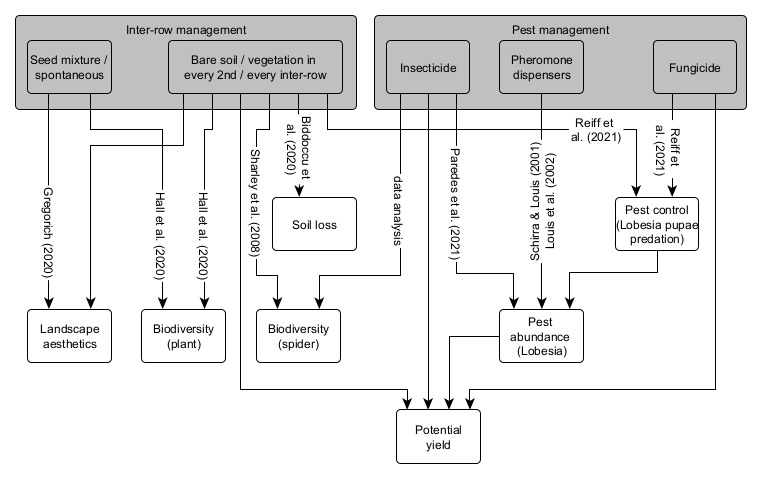
**

**Figure 2. Ecological impacts of inter-row and pest management**

**Key submodels in one time step during the simulation:**

- **implement-behavioural-change**

In this submodel, all winegrowers who are marked with contents in their agent state variable “obligation” due to newly introduced policy, and all winegrowers who are marked with contents in their state variable “intervention” are called to carry out the exact behavioural change as described (at the end of the previous time step). For the first time step in the simulation, there are no behavioural changes but agents will repeat their behaviours as initialised in the setup. For each winegrower, the two relevant agent state variables “my-potential-yield-down” and “my-potential-yield-up” are updated with each behavioural change that has influence on yield potential. The “my-potential-yield-down” records the number of behavioural changes that potentially decrease yield: they are increase in inter-row vegetation cover due to potential competition for water, decrease in insecticides and decrease in fungicides due to higher chances of losing yield due to insects or fungi; likewise, the “my-potential-yield-up” records the number of behavioural changes that potentially increase yield: they are decrease in inter-row vegetation cover, increase in insecticides, adoption of pheromone dispensers, and increases in fungicides.

- **check-weather-and-adaptation**

In this submodel, the current climate conditions as described with temperature change and precipitation change is read. Given the empirical evidence from the questionnaire survey, certain combinations of temperature and precipitation change will trigger the adaptative pesticide use concerning insecticides and fungicides. In general, wetter conditions lead to more fungicide use and/or insecticides, and drier conditions lead to less fungicides and/or insecticides, in combination with warmer temperature. These adaptive behaviours are on-the-fly for the current time step, therefore, are implemented immediately. The agent state variables “my-potential-yield-down” and “my-potential-yield-up” are also updated after the implementation of the adaptive behaviours.

- **update-behaviour-status**

In this submodel, all system-level statistics concerning all winegrowers’ behaviours are updated and recorded in lists that document changes over time.

- **update-ecologicals**

In this submodel, links between winegrowers’ behaviours and their ecological impacts are checked again, given the possible changes in behaviours. The exact sequence of the modules called in the  **report-ecological** (in initialisation) are called again.

- **check-policy**

In this submodel, the relevant winegrowers who are subject to the newly introduced policy are called to change their state variable “obligation” with contents relevant to the policy. The policy which is introduced at a manually chosen time step (a model parameter) and winegrowers will respond to the policy at the next time step in the submodel of **implement-behavioural-change**.

- **check-problems**

In this submodel, the status of three agent state variables “yield-problem”, “soil-problem”, “biodiversity-problem” are checked to be true of false. An agent has a “yield-problem” when (s)he has high economic attitude (“EC-mean” >= 3) and “my-potential-yield-down” - “my-potential-yield-up” >= “threshold-yield-level” (a model parameter); an agent has a “soil problem” when (s)he has high soil preserving attitude (“EN-soil” >= 4) and “my-soil-loss” > “threshold-soil” (a model parameter); an agent has a “biodiversity-problem” when (s)he has high biodiversity preserving attitude (“EN-biodiversity” >= 4) and “my-spider-diversity” < “threshold-biodiversity” ( a model parameter). If multiple problems are true, only one of them with be set as “priority”.

- **check-causes**

In this submodel, the cause of a problem as defined in the state variable “priority” is deliberated, based on the current behaviour and the initial behaviour. After the cause of the problem is identified, the relevant state variables (cause-by-IR, cause-by-insecticide, cause-by-fungicide, need-pheromone) will be set to true accordingly**.**

- **check-options**

In this submodel, the intervention to solve a priority problem is deliberated, based on the cause of the problem, and options are constrained by “management-type” and “obligation” due to policy. The corresponding solution will be recorded in the state variable “intervention” and will be implemented in the next time step.

III.iv.b What are the model parameters, their dimensions and reference values?

See Table 9 .

**Table 9. Model parameters and their reference values**

| **Model parameters** | **Dimensions** | **Reference values** |
| --- | --- | --- |
| “threshold-yield-level” | [-5, 5], difference in number of events that decrease and increase the yield potential | 3 |
| “threshold-soil” | [1, 50], ton/hectare | 15 |
| “threshold-biodiversity” | [1, 4], count of species diversity | 2 |
| “policy-start-at” | [1, 5], time step | 1 |
| “fungicide-reduction” | [1, 4], reduction in annual frequency | 1 |

III.iv.c How were the submodels designed or chosen, and how were they parameterised and then tested?

Design: the design of the submodels is based on empirical evidence, data analysis, literature, interview with winegrowers and expert opinion from case study regions.

Parameterisation: all parameters concerning the ecological impacts of winegrowers’ behaviours are based on the most recent and relevant published studies or data analyses. Parameters that are model design choices (e.g., see Table 4) are tested via one-factor-at-a-time (OFAT) sensitivity analysis.

**References:**

Biddoccu, M., Guzmán, G., Capello, G., Thielke, T., Strauss, P., Winter, S., Zaller, J. G., Nicolai, A., Cluzeau, D., Popescu, D., Bunea, C., Hoble, A., Cavallo, E., & Gómez, J. A. (2020). Evaluation of soil erosion risk and identification of soil cover and management factor (C) for RUSLE in European vineyards with different soil management. *International Soil and Water Conservation Research*, *8*(4), 337–353. https://doi.org/https://doi.org/10.1016/j.iswcr.2020.07.003

Chen, Y., Herrera, R. A., Benitez, E., Hoffmann, C., Möth, S., Paredes, D., Plaas, E., Popescu, D., Rascher, S., Rusch, A., Sandor, M., Tolle, P., Willemen, L., Winter, S., & Schwarz, N. (2022). Winegrowers’ decision-making: A pan-European perspective on pesticide use and inter-row management. *Journal of Rural Studies*, *94*, 37–53. https://doi.org/https://doi.org/10.1016/j.jrurstud.2022.05.021

Corine. (2018). *Corine Land Cover Map 2018*. https://land.copernicus.eu/pan-european/corine-land-cover/clc2018?tab=download

Gregorich, C. A. (2020). *ÄSTHETISCHE BEWERTUNG VON WEINGÄRTEN UND WAHRNEHMUNG DER WEINBAULANDSCHAFT – eine empirische Untersuchung aufgezeigt am Beispiel des Weinbaugebietes*. Universität für Bodenkultur Wien.

Hall, R. M., Penke, N., Kriechbaum, M., Kratschmer, S., Jung, V., Chollet, S., Guernion, M., Nicolai, A., Burel, F., Fertil, A., Lora, Á., Sánchez-Cuesta, R., Guzmán, G., Gómez, J., Popescu, D., Hoble, A., Bunea, C.-I., Zaller, J. G., & Winter, S. (2020). Vegetation management intensity and landscape diversity alter plant species richness, functional traits and community composition across European vineyards. *Agricultural Systems*, *177*, 102706. https://doi.org/https://doi.org/10.1016/j.agsy.2019.102706

Liu, J., Dietz, T., Carpenter, S. R., Folke, C., Alberti, M., Redman, C. L., Schneider, S. H., Ostrom, E., Pell, A. N., & Lubchenco, J. (2007). Coupled human and natural systems. *AMBIO: A Journal of the Human Environment*, *36*(8), 639–649.

Ljungqvist, F. C., Seim, A., Krusic, P. J., González-Rouco, J. F., Werner, J. P., Cook, E. R., Zorita, E., Luterbacher, J., Xoplaki, E., Destouni, G., García-Bustamante, E., Aguilar, C. A. M., Seftigen, K., Wang, J., Gagen, M. H., Esper, J., Solomina, O., Fleitmann, D., & Büntgen, U. (2019). European warm-season temperature and hydroclimate since 850 CE. *Environmental Research Letters*, *14*(8), 084015. https://doi.org/10.1088/1748-9326/ab2c7e

Louis, F., Schmidt-Tiedemann, A., & Schirra, K.-J. (2002). Control of Sparganothis pilleriana Schiff. and Lobesia botrana Den. & Schiff. in German vineyards using sex pheromone-mediated mating disruption. *IOBC/WPRS Bulletin*, *25*, 1–9.

meteoblue. (2022a). *Climate Change Blaj*.

meteoblue. (2022b). *Climate Change Hof am Leithaberge*.

meteoblue. (2022c). *Climate Change Landau*.

Müller, B., Bohn, F., Dreßler, G., Groeneveld, J., Klassert, C., Martin, R., Schlüter, M., Schulze, J., Weise, H., & Schwarz, N. (2013). Describing human decisions in agent-based models – ODD + D, an extension of the ODD protocol. *Environmental Modelling & Software*, *48*, 37–48. https://doi.org/https://doi.org/10.1016/j.envsoft.2013.06.003

Ostrom, E. (2009). A general framework for analyzing sustainability of social-ecological systems. *Science*, *325*(5939), 419–422.

Paredes, D., Rosenheim, J. A., Chaplin-Kramer, R., Winter, S., & Karp, D. S. (2021). Landscape simplification increases vineyard pest outbreaks and insecticide use. *Ecology Letters*, *24*(1), 73–83. https://doi.org/https://doi.org/10.1111/ele.13622

Reiff, J. M., Kolb, S., Entling, M. H., Herndl, T., Möth, S., Walzer, A., Kropf, M., Hoffmann, C., & Winter, S. (2021). Organic farming and cover-crop management reduce pest predation in austrian vineyards. *Insects*, *12*(3), 1–15. https://doi.org/10.3390/INSECTS12030220

Schirra, K. J., & Louis, F. (2001). The combined use of pheromone and insecticide to reduce high population densities of Lobesia botrana Den & Schiff.(Lepidoptera: Tortricidae) in vineyards. *IOBC WPRS BULLETIN*, *24*(7), 121–126.

Schlueter, M., Mcallister, R. R. J., Arlinghaus, R., Bunnefeld, N., Eisenack, K., Hoelker, F., MILNER‐GULLAND, E. J., Müller, B., Nicholson, E., & Quaas, M. (2012). New horizons for managing the environment: A review of coupled social‐ecological systems modeling. *Natural Resource Modeling*, *25*(1), 219–272.

Sharley, D. J., Hoffmann, A. A., & Thomson, L. J. (2008). effects of soil tillage on beneficial invertebrates within the vineyard. *Agricultural and Forest Entomology*.

1. These attitudes and beliefs are: Respond to current weather conditions; Have resources to implement; Follow advice of extension services; Fit with how my vineyard is managed; Have access to vineyards on rainy days; Avoid water/nutrient competition between vines and inter-rows; Have natural enemies against pests; Show environmental commitment; Preserve soil quality; Preserve biodiversity; Consider hedges and trees in the surroundings; Reduce costs; Benefit from subsidy; Have competitive advantage; Reduce risk of yield loss; Meet customer requirement; Comply with regulations; Have a nice vineyard; Follow traditions; Respect health of workers, tourists, neighbours, etc.; Do not feel guilty; Follow the behaviour of other winegrowers. [↑](#footnote-ref-1)
