# Supplementary figures and images for: Exploring Winegrowers’ Behaviours and Ecological Impacts Under Climate Change and Policy Scenarios—Examples from Three European Winegrowing Regions
Source: Environ Manage. 2024 Jan 11;73(4):841–57. doi: 10.1007/s00267-023-01924-8 (PMC10973083; doi:10.1007/s00267-023-01924-8)

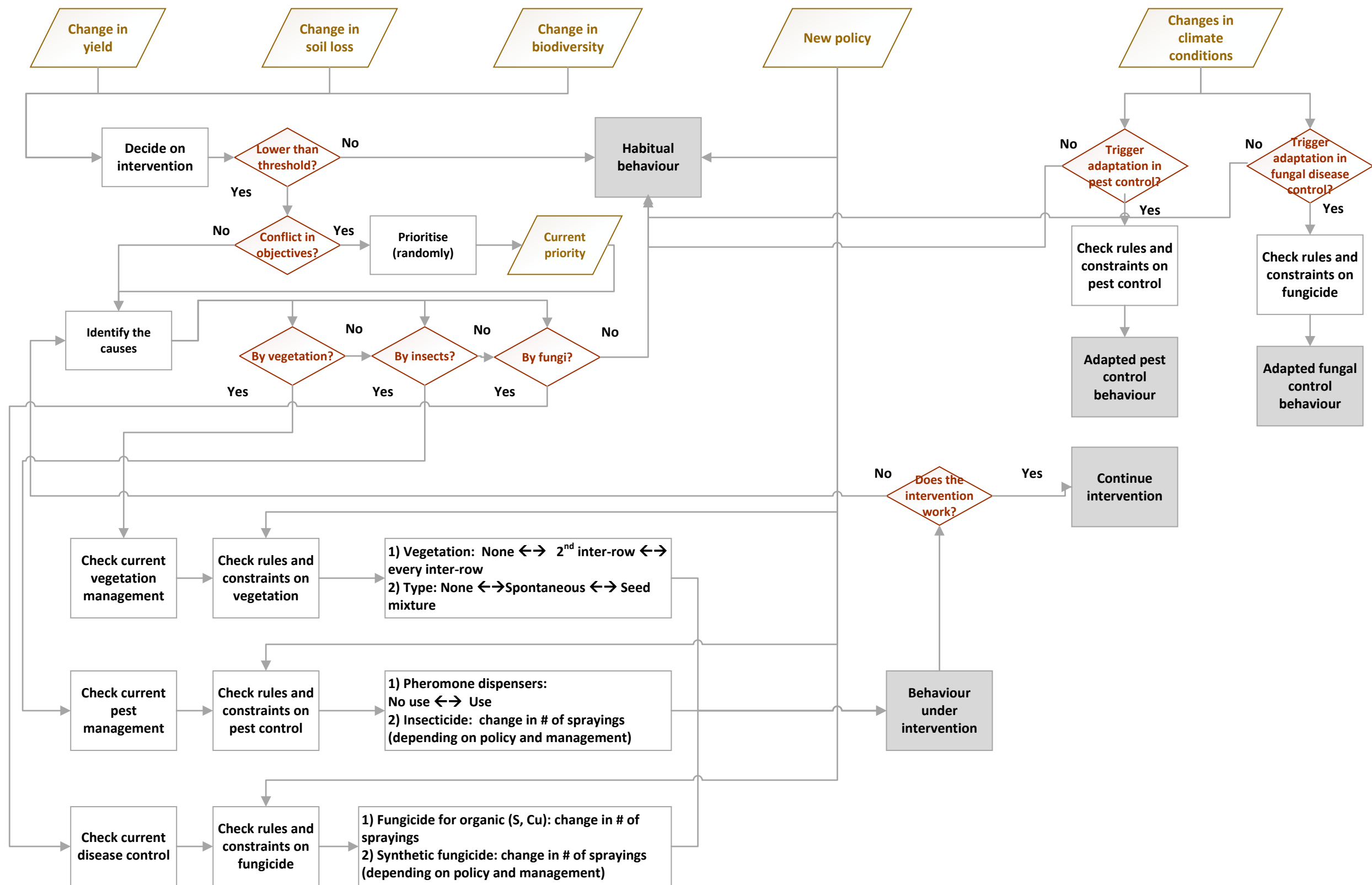

Supplement: Supplementary file 2 — Appendix [file 267_2023_1924_MOESM2_ESM.pdf]
