# Supplementary material for: Exploring Winegrowers’ Behaviours and Ecological Impacts Under Climate Change and Policy Scenarios—Examples from Three European Winegrowing Regions
Source: Environ Manage. 2024 Jan 11;73(4):841–57. doi: 10.1007/s00267-023-01924-8 (PMC10973083; doi:10.1007/s00267-023-01924-8)
Supplement: Supplementary file 3 — Appendix [file 267_2023_1924_MOESM3_ESM.docx]

Appendix 3. Model validation

Model outcomes were qualitatively validated with information from local experts from the research team SECBIVIT. For each case study region, one questionnaire was filled in by the experts. In Romania, the two local experts were scientists at the Department of Environment and Plant Protection, University of Agricultural Sciences and Veterinary Medicine in Cluj-Napoca, one of them is also working as an advisor at a major wine-making company. In Austria, the two local experts were scientists at the Institute of Plant Protection, University of Natural Resources and Life Sciences Vienna, and one of them works also in a wine-growing estate. For Germany, the local expert was a scientist at the Julius Kühn-Institute – Institute for Plant Protection in Fruit Crops and Viticulture. In the two cases where the experts together answered one questionnaire per case study, they agreed on a joint statement beforehand, so that no contradictory statements for the same case study were received.

Table A3.1 Model outcome (in the scenario of no climate change and no new policy) validation against expert estimates. Validation was performed only for winegrowers’ behaviours but not for ecological impacts. Dark grey cells indicate averaged simulation results clearly out of range, while light grey represent averaged simulation results outside but close to the range . *“-” indicates model outcomes that cannot be validated.* ***S indicates simulated mean results****, with the minimal and maximal values in square brackets.* ***E indicates expert estimates, with the minimal and maximal values in square brackets****. Note that our expert from Germany could not provide every estimate due to legal restrictions on reports in the Palatinate area but indicated its similarity to the Leithaberg region.*

| **Type** | **Indicators** | **Leithaberg** | **Palatinate** | **Târnave** |
| --- | --- | --- | --- | --- |
| Winegrowers’ behaviours | Share of vineyards with vegetation in every inter-row (0-1, with 1 being 100%) | S: 0.53 [0.464, 0.589];  E: [0.45, 0.50] | S: 0.61 [0.503, 0.730];  E: NA | S: 0.342 [0.276, 0.388];  E: 0.35 |
|  | Share of vineyards with bare soil only inter-rows | S: 0  E: <= 0.05 | S: 0  E: <= 0.05 | S: 0.175 [0.143, 0.212];  E: 0.23 |
|  | Share of inter-row vegetation as seed mixture | Underestimated  S: 0.52 [0.494, 0.560];  E: 0.85 | Underestimated  S: 0.64 [0.55, 0.73];  E: NA | S: 0  E: 0 |
|  | Mean annual insecticide applications | S: 0  E: for less than 10% of winegrowers with 1 year | S: 0  E: NA | Overestimated  S: 7.11 [6.94, 7.34];  E: 3 |
|  | Share of vineyards using pheromone dispensers | Underestimated  S: 0.576 [0.540, 0.618];  E: 0.90 | S: 1  E: ~ 0.9 | Overestimated  S: 0.626 [0.574, 0.655];  E: 0.25 |
|  | Mean annual application of copper- and/or sulphur-based fungicides | S: 7.29 [7.08, 7.52];  E: [0, 7] | S: 4.31 [3.74, 4.75];  E: NA | S: 4.85 [4.75, 4.96];  E: 5 [4, 7] |
|  | Mean annual application of synthetic fungicides | S: 3.88 [3.75, 3.97];  E: [2,12] | S: 4.93 [4.94, 5.49];  E: NA | S:3.78 [3.73, 3.86];  E: 6 [4, 9] |
| Ecological impacts | Landscape: area extent of vegetated inter-rows (%) | S: 75.7 [72.5, 78.3] | S: 80.7 [75.6, 85.7] | S: 56.0 [40.9, 64.6] |
|  | Mean annual soil loss of vineyards (ton/hectare) | S: 7.35 [6.79, 7.91] | S: 5.19 [4.55, 6.11] | S: 10.31 [9.42, 11.22] |
|  | Mean vascular plant diversity of vineyards | might be underestimated  S:15.3 [15.1, 15.4] | S:16.9 [16.2, 17.7] | S: 12.6 [11.9, 13.1] |
|  | Mean spider diversity of vineyards | S: 8.18 [8.16, 8.20] | S: 8.09 [8.03, 8.13] | might be underestimated  S: 5.14 [5.05, 5.22] |
|  | Mean predation rate of lobesia pupae (%) | S: 85.66 [84.95, 85.96] | S: 85.34 [84.43, 86.90] | S: 92.73 [92.54, 92.87] |
|  | Share of vineyards with high lobesia abundance (0-1) | might be overestimated  S: 0.424 [0.382, 0.461] | S:0 | might be underestimated  S: 0 |
|  | Total times (events) yield potentials are increased per vineyard | - | - | - |
|  | Total times (events) yield potentials are decreased per vineyard | - | - | - |
